# Adaptation to hummingbird pollination is associated with reduced diversification in *Penstemon*

**DOI:** 10.1002/evl3.130

**Published:** 2019-08-02

**Authors:** Carolyn A. Wessinger, Mark D. Rausher, Lena C. Hileman

**Affiliations:** ^1^ Department of Ecology and Evolutionary Biology University of Kansas Lawrence Kansas 66045; ^2^ Department of Biology Duke University Durham North Carolina 27708

**Keywords:** BiSSE, diversification, FiSSE, flower, HiSSE, hummingbird pollination, macroevolutionary equilibrium, *Penstemon*, pollination syndrome

## Abstract

A striking characteristic of the Western North American flora is the repeated evolution of hummingbird pollination from insect‐pollinated ancestors. This pattern has received extensive attention as an opportunity to study repeated trait evolution as well as potential constraints on evolutionary reversibility, with little attention focused on the impact of these transitions on species diversification rates. Yet traits conferring adaptation to divergent pollinators potentially impact speciation and extinction rates, because pollinators facilitate plant reproduction and specify mating patterns between flowering plants. Here, we examine macroevolutionary processes affecting floral pollination syndrome diversity in the largest North American genus of flowering plants, *Penstemon*. Within *Penstemon*, transitions from ancestral bee‐adapted flowers to hummingbird‐adapted flowers have frequently occurred, although hummingbird‐adapted species are rare overall within the genus. We inferred macroevolutionary transition and state‐dependent diversification rates and found that transitions from ancestral bee‐adapted flowers to hummingbird‐adapted flowers are associated with reduced net diversification rate, a finding based on an estimated 17 origins of hummingbird pollination in our sample. Although this finding is congruent with hypotheses that hummingbird adaptation in North American Flora is associated with reduced species diversification rates, it contrasts with studies of neotropical plant families where hummingbird pollination has been associated with increased species diversification. We further used the estimated macroevolutionary rates to predict the expected pattern of floral diversity within *Penstemon* over time, assuming stable diversification and transition rates. Under these assumptions, we find that hummingbird‐adapted species are expected to remain rare due to their reduced diversification rates. In fact, current floral diversity in the sampled *Penstemon* lineage, where less than one‐fifth of species are hummingbird adapted, is consistent with predicted levels of diversity under stable macroevolutionary rates.

**Impact summary**

An important goal of evolutionary biology is to understand processes that determine trait diversity within groups of related species. Flowers adapted to hummingbird pollinators have evolved many independent times in North American flowering plants from insect‐pollinated ancestors, providing an excellent example of convergent evolution. This pattern suggests the evolution of hummingbird‐adapted flowers has often been favored. Little is known about whether transitions to hummingbird pollination impacts species diversification rates such as speciation and extinction rates. We address this issue by examining floral diversification in the largest flowering plant genus endemic to North America, *Penstemon*, which includes nearly 300 species. During diversification, species with hummingbird‐adapted flowers evolved from bee‐adapted ancestors 12–20 times independently, yet hummingbird‐adapted species are relatively rare. Either insufficient time has passed for hummingbird‐adapted species to reach high frequencies within *Penstemon* or perhaps hummingbird pollinated species are kept rare due to reduced diversification rates, such as reduced speciation rate or increased extinction rates. We used a statistical approach to estimate rates of transitioning between floral types along with rates of speciation and extinction associated with each floral type. We found that, despite a high rate of transitioning to hummingbird adaptation, species with hummingbird‐adapted flowers have a lower rate of species diversification. Based on this reduced diversification rate, hummingbird‐adapted species are expected to remain rare over evolutionary time, at a frequency similar to the currently observed level, suggesting that the macroevolutionary effects of pollinator adaptation have been consistent during *Penstemon* diversification.

Across species, certain traits are common while others are rare. Understanding how trait and functional diversity is established and maintained within lineages is a fundamental goal for explaining patterns of biodiversity (Simpson 1953; Losos et al. 1998; Schluter 2000; Wagner et al. 2012). A compelling pattern is parallel evolution where multiple transitions to the same derived state have occurred within a lineage. Given that parallel evolution is often viewed as strong evidence of adaptation, we might expect the derived state to become common within a lineage. Yet in certain cases, the derived state remains relatively rare, despite multiple origins. A classic example of this pattern is the evolution of self‐fertilization in plants (Stebbins 1974; Igić et al. 2008). Other examples include the evolution of host specialization in bark beetles, the evolution of sociality in spiders, and the evolution of asexuality (Kelley and Farrell 1998; Agnarsson et al. 2006; Schwander and Crespi 2009). This pattern of rarity despite high rates of transition to the derived state can lead to a phylogenetic pattern of “tippiness,” as well documented for certain transitions in flower color (Ng and Smith 2018).

In theory, the prevalence of a given derived trait state within a lineage depends on rates of both species diversification and transitions between states, as well as the stability of these processes over time (Maddison 2006; Maddison et al. 2007). If transition and diversification rates are stable over time, the frequency of the derived state will reach an equilibrium value that is predicted from the macroevolutionary rates (Maddison et al. 2007). Therefore, there are at least three explanations for why a derived state might be currently rare. First, the rare state may currently be at or near an equilibrium frequency. Under this explanation, the derived state is rare at equilibrium either because it has a lower diversification rate (speciation rate – extinction rate) than the common state, because there is a higher rate of transitioning from the rare state to the common state than in the reverse direction, or both. We term this the “near equilibrium” explanation. Second, the derived trait may be rare because insufficient time has passed for it to reach its (potentially higher) equilibrium frequency. This explanation does not require differences in diversification rates or asymmetric transition rates. We term this the “approaching equilibrium” explanation. Finally, if transition and/or diversification rates change over time, there is no predicted equilibrium frequency. In this case, the derived state may be rare because current processes favor an alternative trait state, or because there has been insufficient time to reach high frequencies. We term this the “nonequilibrium” explanation.

Although state‐dependent diversification and unequal transition rates between states have frequently been documented (e.g., Tripp and Manos 2008; Goldberg et al. 2010; Beaulieu and Donoghue 2013; Weber and Agrawal 2014; Blanchard and Moreau 2017; Nakov et al. 2017), we are just beginning to understand how these processes combine to explain extant patterns of diversity (O'Meara et al. 2016). One current limitation in distinguishing equilibrium and nonequilibrium explanations for trait rarity is a lack of appropriate tools. Commonly used state‐dependent speciation and extinction (SSE) models that estimate state‐dependent diversification and transition rates assume these rates are stable over time, and implementations of these models have not yet included validated methods for determining whether these rates are temporally stable. Absent such tools, we adopt a different approach by asking whether a near equilibrium explanation is sufficient to account for rare trait frequencies in a phylogeny.

Here, we address this question by examining the impact of floral adaptation to primary pollinator on species diversity in the genus *Penstemon*. Flowers are a key component of functional diversity within angiosperm‐dominated ecosystems (e.g., Barrett et al. 1996; Strauss and Whittall 2006; Stevenson et al. 2017). Floral diversity is driven in part by interactions with pollinators, which lead to divergence in pollination syndromes—multitrait adaptations to a specific pollinating agent (Faegri and Van der Pijl 1979; Fenster et al. 2004). At the genus level, evolutionary transitions in primary pollinator can occur on a rapid timescale and can show asymmetric transition rates (Stebbins 1970; Stebbins 1974; Thomson and Wilson 2008), suggesting selective and developmental constraints influence rates of transitioning to a new pollinator. It is plausible that traits conferring pollinator adaptation influence species diversification rates, given that pollinators facilitate reproduction and specify mating patterns between plants across a landscape. For example, innovative traits associated with increased pollinator specialization, such as bilateral symmetry, floral tubes, and nectar spurs, have been linked to increased diversification rates (Hodges and Arnold 1995; Sargent 2004; Fernández‐Mazuecos et al. 2013; O'Meara et al. 2016; Fernández‐Mazuecos et al. 2018). Consequently, pollinator adaptation that impacts species diversification rates has implications for the maintenance of floral diversity within flowering plant lineages.

Unique feature of the North American Flora is the repeated origin of hummingbird pollination from insect‐pollinated (often bee‐pollinated) ancestors (Stebbins 1989; Grant 1994; Abrahamczyk and Renner 2015). Within this flora, *Penstemon* is the largest endemic flowering plant genus and exhibits a dynamic history of pollination syndrome evolution, with 12–20 transitions from bee or wasp adaptation to hummingbird adaptation, but no obvious reverse transitions (Wilson et al. 2007). Despite this large number of evolutionary origins, hummingbird syndrome species remain rare within *Penstemon*, either because hummingbird syndrome species have a reduced diversification rate, because there is an even higher rate of transitioning from hummingbird back to bee syndrome, or because parallel evolution of hummingbird adaptation is a recent phenomenon and there has not yet been enough time for hummingbird syndrome species to reach high frequencies within the genus. This impressive evolutionary replication makes *Penstemon* an attractive system to test whether pollinator adaptation impacts species diversification rates in North American Flora and whether current trait diversity can be accounted for by a near equilibrium explanation.

To provide a framework for our study, we first updated a phylogenomic dataset to sample over 80% of species in a large clade within *Penstemon*. We then used macroevolutionary analyses to demonstrate that hummingbird‐adapted species have a substantially reduced diversification rate compared to bee‐adapted species. Our analyses were unable to detect unequal transition rates, despite our expectation that transitions from bee‐ to hummingbird‐adapted species have been much more common than reverse transitions. Finally, we used inferred macroevolutionary rate parameters to model the evolution of trait diversity assuming constant rate parameters. This analysis indicates that the current relative proportion of bee‐ versus hummingbird‐adapted species in this *Penstemon* clade, with bee‐adapted species approximately four times as common as hummingbird‐adapted species, is consistent with a near equilibrium explanation.

## Methods

### STUDY SYSTEM

*Penstemon* is a North American flowering plant genus of nearly 300 species. Most species are adapted to pollination by bees or wasps and display a bee pollination syndrome: short and wide corollas, blue or purple color, anthers and stigmas located within the corolla tube, lower petals forming a landing platform for bees, and producing a small amount of concentrated nectar. Over 30 *Penstemon* species are adapted to hummingbird pollination and display a typical hummingbird pollination syndrome: long and narrow corollas, red color, anthers and stigmas exserted outside the corolla tube, lower petals reduced or reflexed, and production of a large amount of dilute nectar (Wilson et al. 2006). These two syndromes form distinct clusters in multidimensional space that correspond to well‐defined bee and hummingbird syndromes that accurately predict pollinator visitation (Wilson et al. 2004, 2006; Wilson and Jordan 2009). A few “despecialized” species show partial adaptation to hummingbird pollination (i.e., large nectar volume and magenta flowers) but retain bees as pollinators (Wilson et al. 2006). Transitions to other pollination syndromes are rare in this genus (Wilson et al. 2007; Thomson and Wilson 2008).

### TAXON SAMPLING AND CHARACTER DATA

Previously we used multiplexed shotgun genotyping (MSG) (Andolfatto et al. 2011) to generate genome‐wide DNA sequence data for phylogenomic analysis of 75 species (Wessinger et al. 2016). Here, we add data for an additional 45 species, yielding a dataset of 120 *Penstemon* species. Our additional sampling was largely focused on the “crown clade” described in Wessinger et al. (2016) (sect. *Ambigui*, sect. *Coerulei*, sect. *Gentianoides*, subg. *Habroanthus*, sect. *Leptostemon*, and sect. *Spectabiles*) because it contains a relatively large number of hummingbird‐adapted species. Our final dataset includes 104 of the presumed 126 species (82.5%) in this clade, with bee and hummingbird syndrome species sampled at similar rates (83% and 79%, respectively). We dichotomized sampled crown clade species into bee syndrome (including despecialized species: *P. bicolor*, *P. floridus*, *P. hartwegii*, *P. isophyllus*, *P. parryi*, and *P. pseudospectabilis*) and hummingbird syndrome (hummingbird specialists) according to Wilson et al. (2007). This strict dichotomization allows us to specifically test the macroevolutionary effect of specialization to hummingbird pollinators. Character state data is found in Table S1. Voucher information for all 120 samples included in the present analysis is deposited in Dryad (https://doi.org/10.5061/dryad.58qg7ct).

### DNA SEQUENCING, PHYLOGENETIC INFERENCE, AND STATE‐INDEPENDENT DIVERSIFICATION ANALYSES

We extracted DNA from new samples, prepared and sequenced MSG libraries, and generated aligned sequence data matrices from raw sequencing reads for the full set of 120 *Penstemon* species (“full dataset”) and the 104 crown clade species (“crown dataset”). For each concatenated dataset, we inferred a maximum likelihood (ML) phylogeny using IQ‐TREE version 1.6.2 (Nguyen et al. 2014) and a species tree using SVDquartets, which models variation in underlying gene trees due to incomplete lineage sorting (ILS; Chifman and Kubatko 2014). Details of these steps follow Wessinger et al. (2016) and are provided in the Supporting Information.

We rescaled crown dataset trees to be ultrametric (proportional to relative time) with a BEAST2 analysis (Bouckaert et al. 2014), constraining the tree topology and constraining the total tree length to 1. We summarized postburnin tree distributions with maximum clade credibility trees that were used in downstream analyses (see Supporting Information for BEAST2 details). In the text, we refer to the ultrametric ML tree as “crown‐ML” and the ultrametric SVDquartets tree as “crown‐SVD.” We also generated a set of trees by artificially truncating the terminal branches of the crown‐ML tree, so that the tree length is reduced by 5%, 7.5%, or 10%, using the treeSlice function in the R package phytools (Revell 2012). We used this set of trees to explore whether inferred terminal branch lengths, which may be inflated by gene tree discordance, affect our hidden state analyses (see Results section). In the main text, we present analyses of the tree with 10% of the length truncated (hereafter “crown‐ML‐cropped”) and present analyses of the 5% and 7.5% truncated trees in the Supporting Information. This truncation necessarily collapsed one pair of taxa (both bee syndrome) into a single tip, which we assigned to bee syndrome.

We quantified patterns of phylogenetic discordance using the quartet concordance (QC) metric (Pease et al. 2018). This metric is the proportion of total taxon quartets having relationships concordant with the given tree topology. We calculated QC values for each node in the crown‐ML and crown‐SVD trees using the quartetsampling program (https://www.github.com/fephyfofum/quartetsampling).

For the crown‐ML and crown‐ML‐cropped trees, we estimated character‐independent patterns of diversification using Bayesian Analysis of Macroevolutionary Mixtures implemented in BAMM version 2.5.0 (Rabosky 2014). Details are found in the Supporting Information.

### STATE‐DEPENDENT DIVERSIFICATION ANALYSES

We fit SSE models to test whether transition rates between pollination syndromes are asymmetric and whether syndrome‐specific diversification rates differ in *Penstemon*. All SSE analyses were restricted to the crown clade trees, where species sampling was high (82.5%). Model parameters include state‐specific speciation rates (λ_0_, λ_1_), state‐specific extinction rates (μ_0_, μ_1_), and transition rates between states (q_01_, q_10_), where state 0 denotes bee syndrome and state 1 denotes hummingbird syndrome. We fit four BiSSE models (Maddison et al. 2007) that differ in their constraints on parameter values. Model B1 has no constraints, model B2 constrains equal transition rates, model B3 prevents transitions from hummingbird to bee syndrome, and model B4 constrains speciation and extinction rates to be equal across pollination syndromes (Table 1). We used the HiSSE framework to additionally model an unobserved character, with states A and B, that potentially impacts species diversification (Beaulieu and O'Meara 2016). We considered three hidden state models. Model CID2 is a character‐independent model where the hidden character, but not the observed character (pollination syndrome), affects diversification rate. Model H1 is a full HiSSE model where both the hidden and observed characters can affect diversification, with no constraints on parameter values. Finally, the CID4 model is similar to CID2, but the hidden character has four states. We provide additional details of these models and our approach in the Supporting Information.

**Table 1:** SSE models analyzed using MCMC in RevBayes for the crown‐ML tree

		Bayes factors relative to competing models						
Model	Marginal lnL	B1	B2	B3	B4	H1	CID2	CID4
B1: r_0_≠r_1_, q_01_≠q_10_	−380.395		−0.01	−0.238	**8.782**	−**4.464**	1.097	−**2.83**
B2: r_0_≠r_1_, q_01_ = q_10_	−380.39	0.01		−0.228	**8.792**	−**4.454**	1.107	−**2.82**
B3: r_0_≠r_1_, q_10_ = 0	−380.276	0.238	0.228		**9.02**	−**4.227**	**1.334**	−**2.592**
B4: r_0_ = r_1_, q_01_≠q_10_	−384.786	−**8.782**	−**8.792**	−**9.02**		−**13.25**	−**7.686**	−**11.612**
H1: r_0A_≠r_1A_≠r_0B_≠r_1B_, q_AB_≠q_BA_, q_01_≠q_10_	−378.163	**4.464**	**4.454**	**4.227**	**13.25**		**5.561**	**1.634**
CID2: r_0A_ = r_1A_≠r_0B_ = r_1B_, q_AB_≠q_BA_, q_01_≠q_10_	−380.943	−1.097	−1.107	−**1.334**	**7.686**	−**5.561**		−**3.926**
CID4: r_0A_ = r_1A_≠r_0B_ = r_1B_≠ r_0C_ = r_1C_≠r_0D_ = r_1D_, 14 transition rates	−378.980	**2.83**	**2.82**	**2.592**	**11.612**	−**1.634**	**3.926**	

Marginal log‐likelihoods (lnL) of each model are given along with pairwise Bayes factor model comparisons. Here, Bayes factors (Bf) are calculated as twice the difference in marginal log‐likelihoods. Values > 1.16 indicate the focal model is preferred over the competing model, and values < –1.16 indicate the competing model is preferred over the focal model. Values satisfying this criterion are in bold. See Table S2 for confidence intervals of inferred parameters.

We generated posterior distributions of parameter values and sampled ancestral character states using MCMC implemented in RevBayes (Höhna et al. 2016) for the crown‐ML, crown‐SVD, and crown‐ML‐cropped datasets. Because estimation of extinction fractions can be prone to bias (Rabosky 2010; Beaulieu and O'Meara 2015; Rabosky 2016), we report net diversification rates (*r*_i_ = *λ*_i_ – *μ*_i_). We compared marginal likelihoods of competing models using Bayes factors, here as twice the difference in marginal log likelihoods (Kass and Raftery 1995). Details of our RevBayes analyses are provided in the Supporting Information.

We used the nonparametric FiSSE statistic (Rabosky and Goldberg 2017) to additionally test for an association between pollination syndrome and diversification rate in the crown‐ML, crown‐SVD, and crown‐ML‐cropped datasets. Details are provided in the Supporting Information.

### TESTS OF MODEL ADEQUACY

We simulated trees under candidate models and compared them to our empirical tree to assess whether a given model is an adequate description of our data (Pennell et al. 2015). For each candidate model, we simulated 1000 trees of 104 species using the R package diversitree (FitzJohn 2012), drawing evolutionary rate parameters from the posterior distributions estimated by RevBayes (see above). For each replicate tree, we calculated four metrics: (1) number of hummingbird syndrome tips, (2) number of hummingbird syndrome tips per origin (Bromham et al. 2015), (3) sum of sister clade differences (measure of phylogenetic signal) (Fritz and Purvis 2010); and (4) tip age rank sum (relative length of hummingbird syndrome tip branches) (Bromham et al. 2015). Additional detail is provided in the Supporting Information. Metrics were calculated using the R package phylometrics (Hua and Bromham 2016). We performed these simulations for models B1, B2, B3, and B4 estimated from the crown‐ML, crown‐SVD, and crown‐ML‐cropped trees.

### EQUILIBRIUM PROPORTIONS OF POLLINATION SYNDROME IN PENSTEMON

We calculated the expected proportions of bee versus hummingbird syndrome species at equilibrium and the time taken to reach equilibrium, assuming stable macroevolutionary rates, under candidate models using a numerical approach. We sampled parameter values for a given model from posterior distributions to obtain a distribution of equilibrium proportions under models B1, B2, B3, and B4 estimated from the crown‐ML, crown‐SVD, and crown‐ML‐cropped trees. Model and simulation details are provided in the Supporting Information.

## Results

### PENSTEMON PHYLOGENOMIC DATA INDICATES SUBSTANTIAL DISCORDANCE

**Figure 1:**
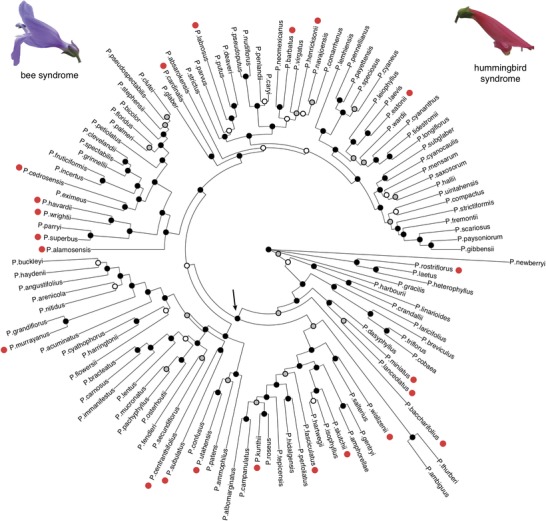
Maximum likelihood tree inferred for the full sample of 120 *Penstemon* species. Hummingbird specialist species are marked with red dots. Nodes are colored according to bootstrap support values: black is greater than 95%, gray is 75–95%, white is less than 75%. Arrow indicates ancestral node to the crown clade. Representative flowers of each pollination syndromes are shown.

The ML and SVDquartets phylogenies for the full dataset of 120 species are fairly congruent, with minor topological differences, and the ML tree has higher bootstrap values than the SVDquartets tree (Fig. 1 and Fig. S1). Both trees show evidence of substantial gene tree discordance according to the calculated QC values (Fig. S2). Such discordance has been found in previous datasets, and has been attributed to a history of ILS and/or hybridization between lineages in *Penstemon* (Wolfe et al. 2006; Wessinger et al. 2016). These population genetic processes are consistent with rapid speciation events and the hypothesis that *Penstemon* represents a continental radiation in North America during the late‐ or post‐Neogene (Wolfe et al. 2006). Given the similarity between the crown‐ML and crown‐SVD trees in terms of topological relationships and diversification rates, we report results of downstream analyses for the crown‐ML tree based on its higher bootstrap and concordance values. All analyses on the crown‐SVD tree produced similar results and are presented in the Supporting Information. Summary information for the aligned datasets and phylogeny concordance values can be found in the Supporting Information.

### BISSE MODELS ASSOCIATE HUMMINGBIRD SYNDROME WITH A REDUCED DIVERSIFICATION RATE

**Figure 2:**
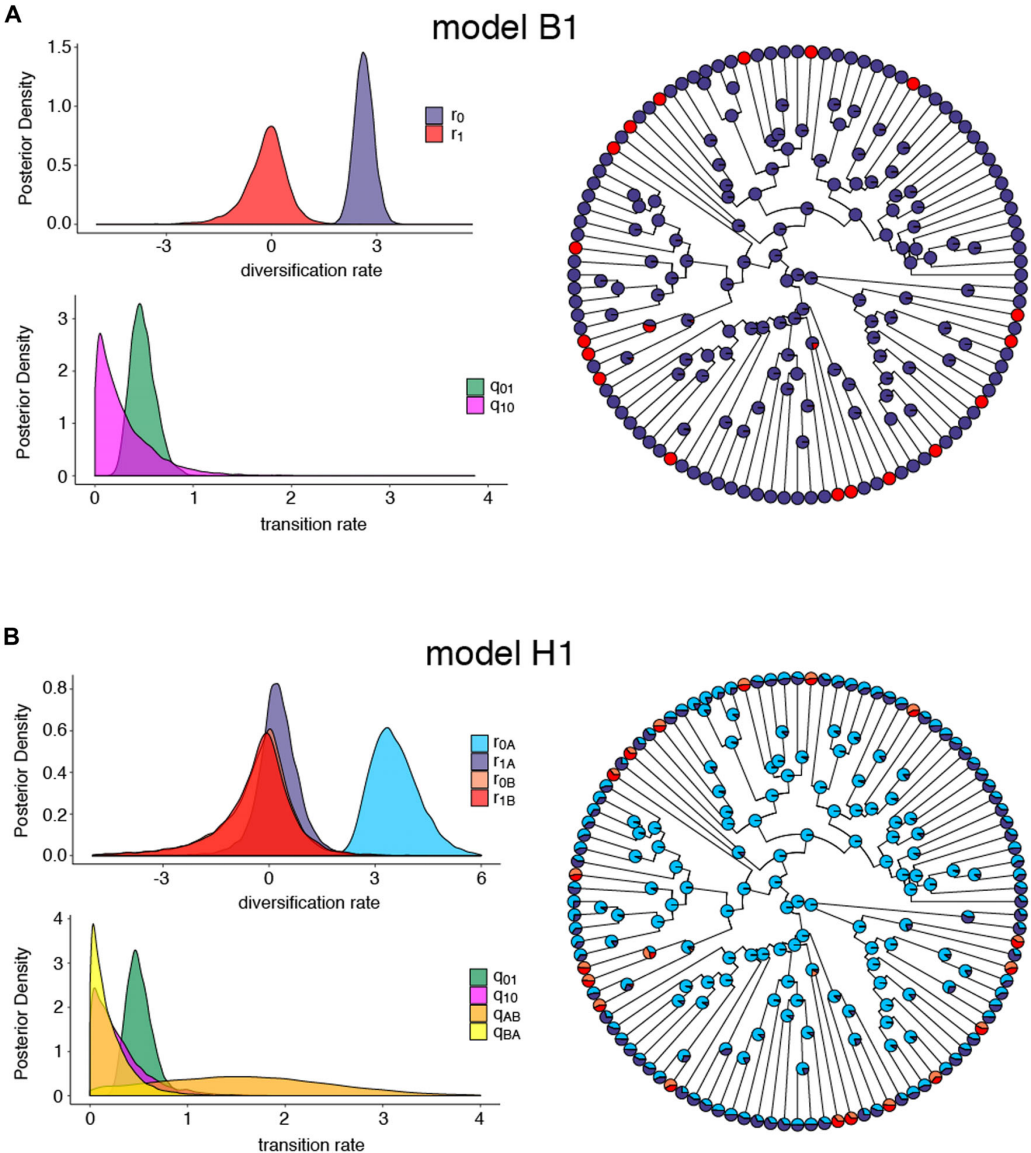
Posterior parameter distributions and ancestral character states for models estimated from the crown‐ML tree. (A) Estimates for full BiSSE model B1 and (B) estimates for full HiSSE model H1. For ancestral states, colors correspond to the posterior probabilities for a given state. In panel A, blue = state 0 (bee syndrome), red = state 1 (hummingbird syndrome). In panel B, light blue = state 0A, light red = state 1A, blue = state 0B, red = state 1B.

Hummingbird syndrome species have a reduced diversification rate relative to bee syndrome species, according to BiSSE analyses of the crown‐ML tree (Fig. 2A; Table S2). This difference is statistically significant, since the unconstrained BiSSE model (model B1) is significantly preferred over the model with equal diversification rates for bee and hummingbird syndrome (model B4; Table 1). The FiSSE analysis on this dataset corroborates this conclusion: the inverse equal splits speciation rate for bee syndrome species is greater than that for hummingbird syndrome species (Λ_0_ = 2.56, Λ_1_ = 2.06), a difference that is significantly larger than expected if diversification rate is not associated with pollination syndrome (*P* = .018).

The BiSSE analyses could not determine whether transition rates are asymmetric. Under the unconstrained model B1, the distributions for transition rates are partially overlapping, with transitions from hummingbird to bee syndrome (q_10_) shifted toward lower values compared with transitions from bee to hummingbird syndrome (Fig. 2A; Table S2). Models that constrained transition rates to be equal (B2) or unidirectional from bee to hummingbird syndrome (B3) were not significantly preferred over the unconstrained model, although the unidirectional model had the highest marginal likelihood (Table 1). The ancestral character reconstruction estimates about 17 transitions to hummingbird syndrome within the sampled species that have occurred near the tips of the tree, with no evidence of reversals from hummingbird to bee syndrome (Fig. 2A). Each origin of hummingbird syndrome includes just one or two species (Fig. 2A). See Figure S3 for the ancestral character reconstruction under the BiSSE model showing taxon names.

A known caveat of the BiSSE model is that trait‐dependent diversification can be incorrectly inferred when diversification rate variation exists that is independent of the modeled trait (Rabosky and Goldberg 2015). In contrast to the BiSSE model, FiSSE rarely implicates trait‐dependent diversification where none exists, even in simulations of complex rate heterogeneity (Rabosky and Goldberg 2017). Therefore, the significant FiSSE result suggests that the BiSSE results are primarily capturing syndrome‐dependent differences in diversification.

### HIDDEN STATE MODELS MAY CAPTURE SYSTEMATIC EFFECT ON TREE SHAPE

The HiSSE modeling framework is designed to overcome false positive results from BiSSE analyses by including a second hidden binary trait that can impact diversification in addition to (or instead of) the observed focal trait. An appropriate null model for comparison to the BiSSE model is the CID2 model that has an equivalent number of diversification rate categories (two) specified by the hidden character, not the observed character. For the crown‐ML tree, the B1 model has a higher marginal likelihood than the CID2 model, although the Bayes factor misses the threshold for model preference (Table 1). The full HiSSE model (model H1) is a better fit than either models B1 or CID2 (Table 1), suggesting both pollination syndrome and a hidden character impact diversification. Alternatively, H1 could be favored over CID2 if our dataset contains rate heterogeneity that is independent of pollination syndrome (Beaulieu and O'Meara 2016; Caetano et al. 2018). Our CID4 model tests this latter possibility. The H1 model is a significantly better fit to the data than the CID4 model (Table 1), supporting the hypothesis that pollination syndrome impacts diversification rate. Interestingly, the CID4 model clearly includes only two diversification rates (Fig. S4), indicating that the most complex CID model for this tree is the CID2 model.

At face value, our HiSSE model results indicate that including a binary hidden character, in addition to pollination syndrome, is a better description of the data than the BiSSE model that only includes pollination syndrome. However, on closer inspection, it appears that the hidden state is likely capturing a systematic effect on branch lengths in our tree. Our study utilized a large genomic dataset to optimize branch lengths in the presence of substantial gene tree discordance. Sources of gene tree discordance such as ILS can artificially elongate terminal branches of a species tree relative to internal branches, a phenomenon termed substitutions produced by ILS (SPILS; Mendes and Hahn 2016). We suspect that the hidden character in our HiSSE analysis is modeling this effect based on the following observations. Nearly all internal nodes are reconstructed as hidden state A (the state with a higher diversification rate) with high probability, whereas tips are equivocally state A or B (Fig. 2B), suggesting hidden state transitions are localized to terminal branches. The transition rate from hidden state B to A (q_BA_) is close to zero (Fig. 2B), consistent with a unidirectional transition from state A to B. Importantly, the posterior distribution of the transition rate from state A to B (q_AB_) is extremely broad (Fig. 2B), suggesting it is a difficult parameter to estimate from the data. Indeed, a systemic effect that increases terminal branch lengths would not be properly modeled as a binary Markov process with transition rates that applies to the entire tree. The ancestral character reconstruction and inferred parameters for model CID2 show similar features (Fig. S5). The observed gene tree discordance (Fig. S2) is consistent with the presence of SPILS in our dataset. In addition, BAMM time‐dependent diversification modeling indicates that diversification rates decline toward the tips of the tree (Fig. S6).

### ARTIFICIALLY CROPPING THE CROWN‐ML TREE REMOVES PREFERENCE FOR HIDDEN STATE MODELS

Given that the hidden state in our HiSSE model may be modeling the SPILs artifact, if we could somehow remove or diminish the effect from our empirical tree, we should lose the clear preference for the hidden state models over the BiSSE model. Our use of MSG data prevented us from applying coalescent‐based species tree approaches to reoptimize branch lengths. Instead, we tested the potential effects of a SPILs artifact on our HiSSE model results by artificially shortening the terminal branches of the crown‐ML tree, so that total tree length is reduced by 10%. This crown‐ML‐cropped tree still displays evidence of syndrome‐specific diversification: the B1 model with reduced diversification rate in hummingbird syndrome species is preferred over the B4 model of equal diversification rates (Table 2). Moreover, the FiSSE statistic still supports differential diversification (Λ_0_ = 3.5, Λ_1_ = 2.67, *P* = 0.03). However, truncating the terminal branches reduced preference of hidden state models such that the B1 model is clearly preferred over the CID2 model and model H1 has a similar marginal likelihood (is no longer clearly preferred) relative to model B1 (Table 2). Under both models, diversification rates are higher for bee syndrome species, while there are no detectable differences among transition rates (Fig. S7, Table S3). In Table S4, we present model preference for analyses conducted on the crown‐ML tree with 5% and 7.5% truncation of tree length. We find that with increasing truncation, we see a gradual loss of preference of hidden state models and increased preference for the B1 model.

**Table 2:** SSE models analyzed using MCMC in RevBayes for the crown‐ML‐cropped tree

		Bayes factors relative to competing models						
Model	Marginal lnL	B1	B2	B3	B4	H1	CID2	CID4
B1: r_0_≠r_1_, q_01_≠q_10_	−353.366		−0.138	−0.498	**9.135**	−0.778	**7.538**	**5.53**
B2: r_0_≠r_1_, q_01_ = q_10_	−353.297	0.138		−0.36	**9.273**	−0.64	**7.676**	**5.668**
B3: r_0_≠r_1_, q_10_ = 0	−353.117	0.498	0.36		**9.633**	−0.28	**8.036**	**6.028**
B4: r_0_ = r_1_, q_01_≠q_10_	−357.934	−**9.135**	−**9.273**	−**9.633**		−**9.913**	−**1.597**	−**3.606**
H1: r_0A_≠r_1A_≠r_0B_≠r_1B_, q_AB_≠q_BA_, q_01_≠q_10_	−352.977	0.778	0.64	0.28	**9.913**		**8.316**	**6.308**
CID2: r_0A_ = r_1A_≠r_0B_ = r_1B_, q_AB_≠q_BA_, q_01_≠q_10_	−357.135	−**7.538**	−**7.676**	−**8.036**	**1.597**	−**8.316**		−**2.008**
CID4: r_0A_ = r_1A_≠r_0B_ = r_1B_≠ r_0C_ = r_1C_≠r_0D_ = r_1D_, 14 transition rates	−356.131	−**5.53**	−**5.668**	−**6.028**	**3.606**	−**6.308**	**2.008**	

Marginal log‐likelihoods (lnL) of each model are given along with pairwise Bayes factor model comparisons. Here, Bayes factors (Bf) are calculated as twice the difference in marginal log‐likelihoods. Values > 1.16 indicate the focal model is preferred over the competing model, and values < –1.16 indicate the competing model is preferred over the focal model. Values satisfying this criterion are in bold. See Table S3 for confidence intervals of inferred parameters.

Together, these results are consistent with our expectation that truncating terminal branch lengths weakens the importance of the hidden state, exposing asymmetry in syndrome‐specific diversification rates. We conclude that the B1 model is a more appropriate model than H1 for modeling syndrome variation in the *Penstemon* crown clade because the hidden state suggested by HiSSE appears to represent a systematic effect on all terminal branches, such as SPILS.

### ESTIMATED MACROEVOLUTIONARY RATES PREDICT EMPIRICAL PROPORTION OF THE HUMMINGBIRD SYNDROME SPECIES

**Figure 3:**
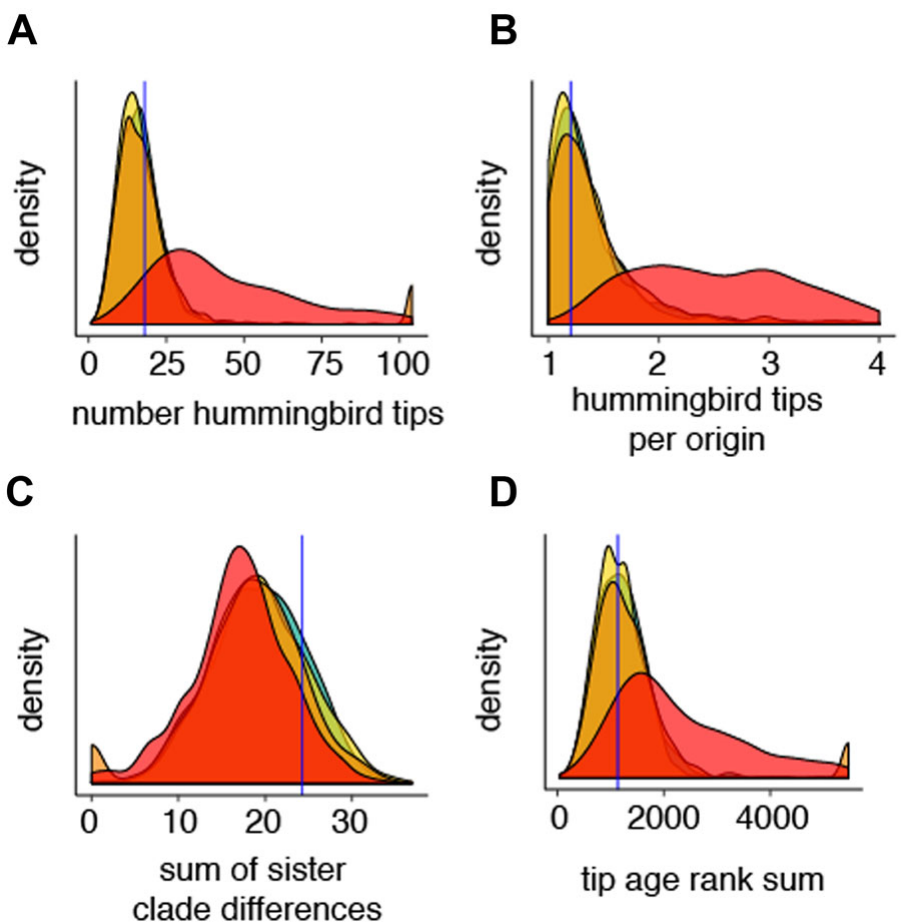
Distributions of metrics for 1000 simulated trees of 104 species for selected models estimated from the crown‐ML tree. Green: model B1 (full BiSSE), yellow: model B2 (equal transition rates), orange: model B3 (unidirectional transitions), and red: model B4 (equal diversification rates). Blue vertical lines indicate observed value for the empirical dataset.

Trees simulated using rates estimated under model B1 for the crown‐ML tree are strikingly similar to the empirical tree according to four metrics: number of hummingbird syndrome tips, number of hummingbird syndrome tips per origin, sum of sister clade differences, and tip age rank sum (Fig. 3). This congruence suggests that the inferred parameter values for the B1 model adequately predict the distribution of hummingbird syndrome in the *Penstemon* crown clade. Trees simulated under parameters estimated for models B2 and B3 display these same patterns, indicating that asymmetric transition rates do not strongly contribute toward the phylogenetic distribution of hummingbird syndrome (Fig. 3). Trees simulated under model B4 estimated parameters have significantly more hummingbird syndrome tips per origin than the observed value of 1.2 (95% confidence interval = 1.43–97.0), indicating that model B4 is unable to predict the characteristic “tippy” distribution of hummingbird syndrome species in the *Penstemon* crown clade.

### HUMMINGBIRD SYNDROME SPECIES ARE RARE AT MACROEVOLUTIONARY EQUILIBRIUM

**Figure 4:**
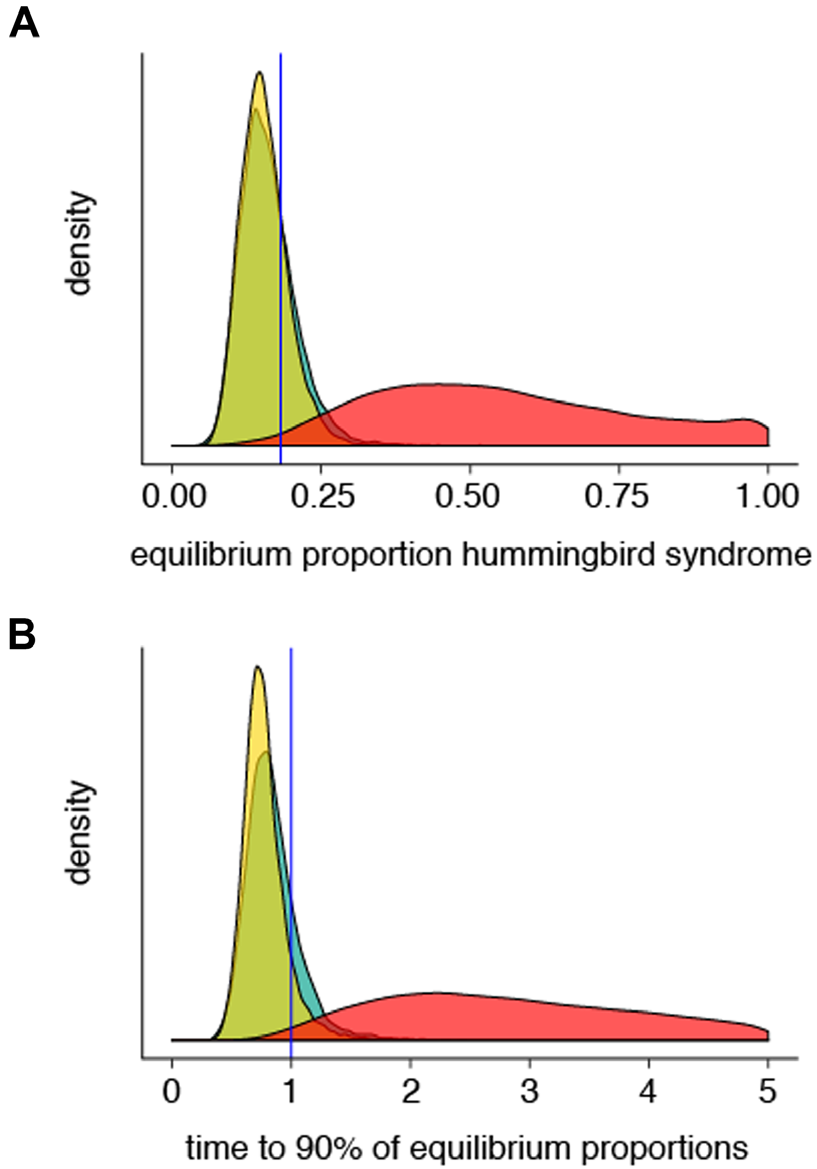
Results from equilibrium calculations for the crown‐ML tree. (A) Distributions of the proportion of hummingbird syndrome species at equilibrium, blue vertical line indicates current proportion of hummingbird syndrome species in the crown clade. (B) Distributions of the time taken for the proportion hummingbird syndrome species to reach 90% of the expected equilibrium proportion. Green: model B1 (full BiSSE), yellow: model B2 (equal transition rates), and red: model B4 (equal diversification rates).

Based on the estimates of macroevolutionary parameters from model B1 for the crown‐ML tree, the expected equilibrium proportion of hummingbird syndrome species in the *Penstemon* crown clade is substantially less than 0.5 (Fig. 4A). The observed proportion of hummingbird syndrome species (0.183) falls within the confidence intervals for this prediction. Moreover, the expected time needed to reach 90% of equilibrium proportions is roughly one time unit (the time taken from the root to the tips of the crown‐ML tree) (Fig. 4B). The distribution of expected proportion of hummingbird syndrome species at equilibrium under the B2 model, where transition rates are equal, does not differ substantially from that calculated for the B1 model—both distributions are centered on 0.16 (Fig. 4A). By contrast, the corresponding distribution calculated for the B4 model, where diversification rates are equal, is broad and is centered on 0.5 (Fig. 4A). This result indicates that, within the context of our data, differential diversification rates have a greater impact on skewing syndrome proportions away from equality than asymmetric transition rates.

## Discussion

### POLLINATION SYNDROME IMPACTS SPECIES DIVERSIFICATION RATE

*Penstemon* is a continental radiation and the sources of diversification rate variation that have shaped its history are likely to be complex, including organismal traits, differences in geographic range, and environmental or geological events. Our study necessarily simplifies over this complexity to examine how pollination syndrome in particular impacts diversification. Our BiSSE, FiSSE, and HiSSE analyses indicate that diversification rates in *Penstemon* are substantially higher for bee‐pollinated species than for hummingbird‐pollinated species. We attribute our power to detect differential diversification in the face of other potential sources of diversification rate variation to the large number of trait origins in our dataset: an estimated 17 origins of the hummingbird syndrome within the crown clade of *Penstemon*.

Previous comparative phylogenetic surveys have suggested a potential association between hummingbird adaptation and reduced diversification rates in North America based on the observation that clades of hummingbird‐adapted species are rare and individual hummingbird‐adapted species are often sister to clades of bee‐adapted species (Abrahamczyk and Renner 2015). One exception is the genus *Castilleja*, where a hummingbird syndrome lineage has diversified into a species‐rich clade (Tank and Olmstead 2008). By contrast, reduced diversification rates associated with hummingbird pollination have not been observed in neotropical groups (e.g., Givnish et al. 2014; Lagomarsino et al. 2017; Serrano‐Serrano et al. 2017), where hummingbird pollination has sometimes been associated with *increased* diversification rates.

This discrepancy may result from the substantially greater hummingbird diversity in the neotropics than in temperate North America, allowing for niche differentiation among bird species to drive plant species diversification. By contrast, hummingbird taxa in North America are not functionally diverse (all species having relatively short and straight bills) relative to bee pollinators that vary in morphology, size, and behavior (e.g., Wilson et al. 2004). Furthermore, differences in hummingbird behavior between the two regions may differentially affect patterns of pollen dispersal (Schmidt‐Lebuhn et al. 2007, 2019). Unlike most tropical hummingbird pollinators, the majority of North American hummingbird species migrate seasonally over large distances, potentially causing substantial gene flow across the landscape relative to bee pollinators (Kramer et al. 2011). This could in theory reduce allopatric speciation rates in hummingbird‐pollinated plant species.

### TRANSITION RATE ASYMMETRY MAY EXIST BUT IS NOT STRONGLY SUPPORTED

There are an estimated 12–20 shifts from bee to hummingbird pollination in *Penstemon*, but no obvious cases of the reverse transition. Moreover, there are ecological and genetic hypotheses for why shifts from bee to hummingbird adaptation may be directionally biased in North American flora (Thomson and Wilson 2008; Barrett 2013; Wessinger and Rausher 2015). However, our analyses were unable to rule out symmetric transitions in any of our analyses, mainly because the transition rate away from hummingbird pollination was so poorly informed by our dataset (no lineage in the data appears to have a long history of hummingbird pollination). We do note that in roughly 80% of the posterior distributions of transition rates illustrated in Figure 2A, the rate of transitioning to hummingbird pollination is greater than the rate of reversals back to bee pollination. Therefore, there is no evidence that reverse transitions to bee adaptation occur at a higher rate than forward transitions to hummingbird adaptation, which means that a high reverse transition rate is unlikely to contribute to the rarity of hummingbird syndrome species.

### OBSERVED PHYLOGENETIC PATTERN IS CONSISTENT WITH MACROEVOLUTIONARY PROCESSES NEAR EQUILIBRIUM

Here, we found that the current observed proportion of bee and hummingbird syndrome species is very near the predicted equilibrium proportions obtained by assuming that macroevolutionary rates are stable over time. Although this agreement of observation with expectation could be an unlikely coincidence, it is also consistent with the near equilibrium hypothesis that macroevolutionary rates in *Penstemon* are relatively stable, and that sufficient time has passed to allow the proportion of hummingbird species to have reached its predicted evolutionary equilibrium. However, we cannot at present definitively rule out the possibility that macroevolutionary rates increase or decrease over time (nonequilibrium explanation), perhaps in a state‐specific manner, because methods for assessing this possibility are not yet available. Nevertheless, it is clear that at least part of the reason hummingbird pollinated species are currently rare is that their diversification rate is lower than that of bee‐pollinated species.

If it is the case that *Penstemon* has achieved macroevolutionary equilibrium for pollination syndrome diversity, then hummingbird syndrome *Penstemon* species should remain substantially less common than bee syndrome species as long as syndrome‐specific diversification rates remain relatively stable in the future. In addition, this scenario would indicate that macroevolutionary equilibrium can be achieved relatively rapidly for labile, ecologically relevant traits. This outcome contrasts with the results of O'Meara et al. (2016), who found that several floral traits in angiosperms as a group are far from their expected macroevolutionary equilibrium. This contrast suggests that, in general, time to reach equilibrium is likely to depend on the trait examined as well as on the taxon and ecological context. All of these unique features can influence both the amount of time that has been available for diversification, as well as the diversification and transition rates.

Associate Editor: L. Bromham

## Supporting information


**Table S1**. Character data used in state‐dependent diversification analyses on crown clade.
**Table S2**. SSE models analyzed using MCMC in RevBayes for the crown‐ML tree.
**Table S3**. SSE models analyzed using MCMC in RevBayes for the crown‐ML‐cropped tree.
**Table S4**. Marginal log‐likelihoods (lnL) of selected SSE models estimated for the crown‐ML tree with 5% and 7.5% of total length truncation.
**Table S5**. SSE models analyzed using MCMC in RevBayes for the crown‐SVD tree. Marginal loglikelihoods (lnL) of each model are given along with estimates of diversification rates (*r*) and transition rates (*q*).
**Fig. S1**. Species tree inferred for the full sample of 120 Penstemon species using SVDquartets.
**Fig. S2**. Distributions of quartet concordance metric values calculated for each node.
**Fig. S3**. Ancestral character states for the crown‐ML tree under the B1 BiSSE model, showing taxonnames.
**Fig. S4**. Posterior densities of net diversification rates for the CID4 model estimated from crown‐ML tree.
**Fig. S5**. Posterior distributions of parameter values and ancestral character states under the CID2 model estimated from crown‐ML tree.
**Fig. S6**. Character‐independent patterns of diversification in the Penstemon crown clade.
**Fig. S7**. Posterior parameter distributions and ancestral character states for models estimated from the crown‐ML‐cropped tree.
**Fig. S8**. Posterior parameter distributions and ancestral character states for models estimated from the crown‐SVD tree.
**Fig. S9**. Distributions of metrics for 1000 simulated trees of 104 species for selected models estimated from the crown‐SVD tree.
**Fig. S10**. Results from equilibrium calculations for the crown‐SVD tree.
