# Supplementary material for: Adaptation to hummingbird pollination is associated with reduced diversification in Penstemon
Source: Evol Lett. 2019 Aug 2;3(5):521–33. doi: 10.1002/evl3.130 (PMC6791294; doi:10.1002/evl3.130)
Supplement: Supplementary file 1 — Table S1. Character data used in state‐dependent diversification analyses on crown clade. Table S2. SSE models analyzed using MCMC in RevBayes for the crown‐ML tree. Table S3. SSE models analyzed using MCMC in RevBayes for the crown‐ML‐cropped tree. Table S4. Marginal log‐likelihoods (lnL) of selected SSE models estimated for the crown‐ML tree with 5% and 7.5% of total length truncation. Table S5. SSE models analyzed using MCMC in RevBayes for the crown‐SVD tree. Marginal loglikelihoods (lnL) of each model are given along with estimates of diversification rates (r) and transition rates (q). Fig. S1. Species tree inferred for the full sample of 120 Penstemon species using SVDquartets. Fig. S2. Distributions of quartet concordance metric values calculated for each node. Fig. S3. Ancestral character states for the crown‐ML tree under the B1 BiSSE model, showing taxonnames. Fig. S4. Posterior densities of net diversification rates for the CID4 model estimated from crown‐ML tree. Fig. S5. Posterior distributions of parameter values and ancestral character states under the CID2 model estimated from crown‐ML tree. Fig. S6. Character‐independent patterns of diversification in the Penstemon crown clade. Fig. S7. Posterior parameter distributions and ancestral character states for models estimated from the crown‐ML‐cropped tree. Fig. S8. Posterior parameter distributions and ancestral character states for models estimated from the crown‐SVD tree. Fig. S9. Distributions of metrics for 1000 simulated trees of 104 species for selected models estimated from the crown‐SVD tree. Fig. S10. Results from equilibrium calculations for the crown‐SVD tree. [file EVL3-3-521-s001.pdf]

Hummingbird adaptation is associated with reduced diversification in *Penstemon*

Supplemental Information

1. Supplemental methods.

DNA sequencing

We extracted DNA following a modified CTAB protocol and prepared MSG libraries using the restriction enzyme *NdeI*, exactly as described by (Wessinger et al. 2016). The new samples were multiplexed using unique barcoded adapters and sequenced in two batches, on the equivalent of one Illumina HiSeq 2500 lane at the University of Kansas Genome Sequencing Core. We have deposited new sequence data in the NCBI Sequence Read Archive (PRJNA548140). Sequence data for samples from our earlier study (Wessinger et al. 2016) is was previously deposited in the SRA (PRJNA314392).

We generated aligned sequence datasets from raw sequencing reads using ipyrad v0.7.22 (https://github.com/dereneaton/ipyrad). Reads were first demultiplexed according to barcodes, allowing one mismatch in barcode sequences. We then trimmed adapter reads using the cutadapt algorithm implemented in ipyrad (filter_adapters = 2). Reads were additionally trimmed (to a minimum length of 35 bp) if they contained low quality bases and reads were discarded if more than 5 bases had phred quality scores less than 20. We enabled reverse complement clustering by setting datatype to 'gbs'. We then clustered reads de novo within individuals using a minimum clustering threshold of 0.90 (90% similarity), the clustering threshold previously used for phylogenomic analyses in *Penstemon* (Wessinger et al. 2016). The minimum read depth was set to 6, the maximum number of uncalled bases per consensus sequence was set to 5, and the maximum alleles per consensus sequence per individual was 2 (all included species are presumed diploid). Sequences were then clustered across individuals using a clustering threshold of 0.90. Consensus loci were aligned if there were at least 20 taxa per locus, no more than 50 SNPs and 8 indels per locus, and no more than 8 shared heterozygous sites across samples. We generated two aligned data matrices. The first includes the full set of 120 *Penstemon* species ('full dataset'). The second includes the 104 crown clade *Penstemon* species ('crown dataset').

The aligned datasets are relatively sparse due to our low threshold for minimum taxa per locus. This allows homologous loci to be sampled, even in the face of large amounts of missing data. Causes of missing data include mutational disruption of restriction enzyme cut sites and stochasticity due low and uneven sequencing coverage. Even datasets with large amounts of missing data can be phylogenetically informative, particularly as the number of included samples increases (e.g., Huang and Knowles 2014; Eaton et al. 2017).

Phylogeny inference

We used IQ-TREE v1.6.2 (Nguyen et al. 2014) to infer a maximum likelihood (ML) phylogeny for each aligned and concatenated dataset. IQ-TREE performs favorably compared to other fast ML-based phylogenetic software (Zhou et al. 2017). Specifically, IQ-TREE is less prone to become trapped in local optima because it maintains a set of candidate trees during the analysis. We performed ten independent IQ-TREE analyses for each dataset using a GTR + Γ model of nucleotide substitution, 1000 ultrafast bootstrap approximations (Hoang et al. 2017), and otherwise default settings. We used the highest-likelihood tree of the ten replicate analyses for downstream analyses.

In addition, we estimated a species tree for each dataset using SVDquartets (Chifman and Kubatko 2014), a species tree approach that accounts for incomplete lineage sorting, implemented in Paup* v4.0a161 (Swofford 2003). We sampled 100,000 quartets from the data matrix and assessed support using 200 bootstrap replicates. SVDquartets does not currently estimate branch lengths, therefore we optimized branch lengths for the SVDquartets topology using a constrained tree search in IQ-TREE under a GTR + Γ model.

We rooted phylogenetic trees for the full dataset on the branch leading to *P. newberryi* (Wolfe et al. 2006) and we rooted trees for the crown dataset on the internal branch separating members of subg. *Leptostemon* from other members, a consistent relationship identified in the full dataset ML tree and in our previous phylogenomic study (Wessinger et al. 2016).

BAMM analysis

For the crown-ML and crown-ML-cropped trees, we estimated character-independent patterns of diversification using Bayesian Analysis of Macroevolutionary Mixtures implemented in BAMM v2.5.0 (Rabosky 2014). BAMM is designed to detect diversification rate heterogeneity across lineages and through time. We set the global sampling fraction to 0.8254 and the expected number of diversification rate shifts to 1. For each tree, we ran two replicate MCMC chains of 1 million generations, sampling every 100 generations, and assessed convergence and stationarity using Tracer (Rambaut et al. 2018). We analyzed results using the R package BAMMtools (Rabosky et al. 2014) to find the best model of shift configurations using Bayes factor model comparison.

Generation of ultrametric trees

We generated ultrametric trees for the crown dataset ML tree and the crown dataset SVDquartets tree using BEAST2 v2.5.1 (Bouckaert et al. 2014) to estimate relative divergence times on a constrained topology. We included all sites in a single partition and used a Γ site model with a GTR substitution model. We used a relaxed normal distribution clock model and a Yule model prior on diversification. For each of these analyses, we performed four MCMC chains, each consisting of 50,000,000 generations, sampling every 50,000 generations. We ran analyses on CIPRES (Miller et al. 2010). We discarded the first 10% of chains as burnin and combined replicate runs. We ensured combined sample reached stationarity using Tracer v1.7.1 (Rambaut et al. 2018) and used TreeAnnotator to exported Maximum Clade Credibility trees with median node heights for downstream analyses.

State-dependent diversification analyses implemented in RevBayes

We tested our prediction that transitions between pollination syndromes in *Penstemon* are asymmetric and that hummingbird syndrome lineages have reduced diversification rates by fitting state-dependent speciation and extinction (SSE) models. SSE models are designed to detect the effect of a trait on lineage diversification using a likelihood framework that jointly models character evolution and character-dependent birth-death processes. Model parameters include state-specific speciation rates (λ_i), state-specific extinction rates (μ_i), and transition rates between states (q_{ij}).

The Binary State Speciation and Extinction model (BiSSE) model considers whether a single observed character potentially affects diversification rate. We fit a series of four BiSSE models with states 0 (bee syndrome) and 1 (hummingbird syndrome). Model B1 has no constraints on parameter values, allowing the six BiSSE model parameters to vary ($\lambda_0 \neq \lambda_1$, $\mu_0 \neq \mu_1$, $q_{01} \neq q_{10}$). Model B2 constrains transition rates

to be equal ($q_{01} = q_{10}$). Model B3 does not allow transitions from hummingbird to bee syndrome ($q_{10} = 0$). Finally, model B4 is the null model of a single diversification rate that constrains speciation and extinction rates to be equal ($\lambda_0 = \lambda_1$, $\mu_0 = \mu_1$).

In addition to these BiSSE analyses, we performed Hidden State Speciation and Extinction (HiSSE) (Beaulieu and O'Meara 2016) analyses. The BiSSE model assumes no additional sources of diversification rate heterogeneity beyond the modeled binary character (Maddison et al. 2007). However, diversification rate variation across the tree may be independent of the observed character and instead be driven by another unmeasured biological character that is nested or overlapping with the observed character. In this case, the BiSSE model will usually be preferred over a "null" model of a single diversification rate regime (Rabosky and Goldberg 2015). To avoid a spurious conclusion of character-dependent diversification, an appropriate null model for comparison to a BiSSE model is a character-independent model with two diversification rate categories (CID2), specified by an unobserved binary hidden character (Beaulieu and O'Meara 2016). Similarly, a full HiSSE model, where the observed character and a hidden character can each affect diversification rate (four diversification rate categories), should be compared to the character-independent model with four diversification rate categories (CID4).

The CID2 and HiSSE models include an unobserved character, with states A and B, that can additionally impact diversification. Model parameters include state-specific speciation and extinction rates for the four possible character-state combinations (0A, 1A, 0B, 1B) and transition rates between the four states. Here we used four transition rates (q_{AB}, q_{BA}, q_{01}, and q_{10}) that allow transition rates in a given character to be independent of the other character, except that simultaneous transitions in both characters are not possible. We considered three hidden state models. The first is a character-independent model where the hidden character, but not the observed character, affects diversification rate (CID2 model: $\lambda_{0A} = \lambda_{1A}$, $\lambda_{0B} = \lambda_{1B}$, $\mu_{0A} = \mu_{1A}$, $\mu_{0B} = \mu_{1B}$). We also fit a full HiSSE model where both the hidden and observed characters can affect diversification (model H1), with no constraints on parameter values. Finally, we fit a character-independent model where the hidden character has four states (A-D), leading to eight character state combinations (0A, 1A, 0B, 1B, 0C, 1C, 0D, 1D). This CID4 model has 14 transition rates (q_{AB}, q_{AC}, q_{AD}, q_{BA}, q_{BC}, q_{BD}, q_{CA}, q_{CB}, q_{CD}, q_{DA}, q_{DB}, q_{DC}, q_{01}, and q_{10}) that are the natural expansion of the transition rates found in our CID2 and HiSSE models.

We generated posterior distributions of parameter values and sampled ancestral character states using MCMC implemented in RevBayes (Höhna et al. 2016) for the crown-ML and crown-ML-cropped datasets. We set lognormal prior distributions for speciation and extinction rates, where log-speciation and log-extinction were distributed with mean = 0 and standard deviation = 1. We set exponential prior distributions for transition rates with mean = 15 transitions / total treelength. We accounted for incomplete sampling by setting the probability of sampling species in the crown clade to 104/126. We note that we have sampled bee vs. hummingbird syndrome species in the crown clade at similar rates (83.5% of bee syndrome species and 78.2% of hummingbird syndrome species). Each MCMC chain drew 20,000 samples from the posterior distribution and we discarded the first 20% of samples as burn-in. We sampled ancestral character states during the MCMC in order to visualize estimates of the ancestral states by plotting posterior probabilities for each state at each node. We checked stationarity using Tracer to ensure ESS values for all parameters were above 200. The CID4 model did not reach stationarity after 20,000 generations, so we ran 40,000 generations for this model, at which point it reached stationarity.

We compared models by estimating the marginal likelihoods of competing models and comparing their relative fit to the data using Bayes factors. We estimated marginal likelihoods using the stepping-stone algorithm implemented in RevBayes, which estimates the marginal likelihood of a given model using a series of MCMC-like simulations that iteratively sample likelihood space between the prior and posterior distributions. We refer the reader to the RevBayes manual for additional information on the stepping-stone algorithm (Höhna et al. 2017). We sampled from 50 steps between the prior and posterior

probability distributions. For each step, we sampled 10,000 generations from the posterior distribution after discarding 10,000 as burnin. We calculated Bayes factor as twice the difference in marginal log likelihoods, using Bayes Factor = 1.16 as a significance threshold for model preference (Kass and Raftery 1995), as recommended in the RevBayes manual (Höhna et al. 2017).

FiSSE statistic calculations

We used the non-parametric Fast, intuitive State-dependent Speciation and Extinction (FiSSE) statistic (Rabosky and Goldberg 2017) to test for an association between pollination syndrome and diversification rate in the crown-ML and crown-ML-cropped datasets. FiSSE computes the average inverse equal splits measure for each character state (Λ_i), a metric that is correlated with, but not equal to, a tip-specific speciation rate. It then compares the observed difference in these values ($\Lambda_1 - \Lambda_0$) to a distribution of values simulated under a model where diversification rate is uncorrelated with the focal trait. We implemented FiSSE on the crown-ML and crown-ML-cropped datasets with the R FISSE.binary function (Rabosky and Goldberg 2017), using 10,000 simulations to test significance.

Additional information on metrics calculated for simulated trees

We calculated a set of metrics on our simulated trees to assess the degree of tippiness for our derived trait of hummingbird-adapted flowers. These metrics are well-described by Bromham et al. (2015), and we briefly describe them here. Tip age rank sum (TARS) represents the rank sum of terminal branches with the derived state and provides a relative measure of the ages of species with the derived state relative to those with the ancestral state. Number of tips per origin (NoTO) calculates the number of tips per origin, as determined through parsimony-based reconstruction, and provides a metric for the degree of tippiness of trait origins. Sum of sister-clade differences (SSCD) sums, for each node in the tree, the number of times the descendent sister clades differ in the modeled binary trait, providing a metric of phylogenetic clustering.

Simulated equilibrium proportions of bee vs. hummingbird syndrome species in Penstemon

Here we describe our methods for numerically calculating equilibrium proportions of bee vs. hummingbird syndrome species. In practice, this method produces effectively identical results as the equations presented in Appendix 2 of Maddison et al. (2007), yet allows us to calculate the time taken to reach equilibrium proportions.

The estimated parameters for BiSSE analyses are:

r_0 = diversification rate for bee syndrome species
r_1 = diversification rate for hummingbird syndrome species
q_{01} = transition rate from bee to hummingbird syndrome
q_{10} = transition rate from hummingbird to bee syndrome

These parameters correspond to the rate of change over a unit time of 1.0, the total tree length. For simulations, we converted these rate parameters into approximations of instantaneous rates by considering time intervals of length 0.001. We performed the following transformations to obtain these instantaneous rate parameters:

$$r_0^* = 1 + r_0/1000$$

$$r_1^* = 1 + r_1/1000$$
$$q_{01}^* = q_{01}/1000$$
$$q_{10}^* = q_{10}/1000$$

Since the diversification rates r_i represent the net rate at which additional species are added (or subtracted), we must add 1 to the instantaneous rates to represent the current number of species.

With these quantities, two matrices are constructed. The first is the diversification matrix R:

$$R = \begin{bmatrix} r_0^* & 0 \\ 0 & r_1^* \end{bmatrix}$$

and the second is the transition matrix, T:

$$T = \begin{bmatrix} 1 - q_{01}^* & q_{10}^* \\ q_{01}^* & 1 - q_{10}^* \end{bmatrix}$$

The current number of species at time t that are bee syndrome (state 0) and hummingbird syndrome (state 1) are represented by the vector N_t:

$$N_t = \begin{bmatrix} N_0 \\ N_1 \end{bmatrix}$$

To find the species numbers in the next time step, we multiply N_t by their diversification rates and by their transition rates:

$$N_{t+1} = RTN_t = \begin{bmatrix} r_0^* & 0 \\ 0 & r_1^* \end{bmatrix} \begin{bmatrix} 1 - q_{01}^* & q_{10}^* \\ q_{01}^* & 1 - q_{10}^* \end{bmatrix} \begin{bmatrix} N_0 \\ N_1 \end{bmatrix}$$

The species numbers after two time steps is found by:

$$N_{t+2} = RTN_{t+1} = RTRTN_t = (RT)^2 N_t$$

More generally, after t time units:

$$N_{t=t} = (RT)^t N_0$$

The equilibrium proportions of the two species are then given by the eigenvector of the matrix:

$$M = RT$$

corresponding to the largest eigenvalue of M, where the elements of the eigenvector are rescaled to sum to 1. We calculated these proportions for the posterior distribution of parameter values, after discarding the first 20% of values as burnin. From the distribution of proportions, we calculated the mean, 95% credible intervals, and 99% credible intervals.

We examined the time taken to approach equilibrium proportions by letting $N_{t=0} = \begin{bmatrix} 1 \\ 0 \end{bmatrix}$, starting with a single ancestral bee syndrome species, and calculating N_t until the proportion of hummingbird syndrome species reaches 90% and 95% of the expected equilibrium proportion. Again, this analysis was performed

for all parameter combinations in the post-burnin posterior distributions. From these calculated distributions of times taken to reach 90% and 95% of equilibrium proportions, we calculated means and 95% credible intervals.

2. Supplemental Results

Features of aligned sequence datasets

Adding 45 new samples to our phylogenomic dataset (Wessinger et al. 2016) yielded concatenated alignments for the full dataset of 120 species and for the crown dataset of 104 species. The full dataset alignment included 2306 loci present in at least 20 species, with 72% missing data. The crown dataset included 2051 loci in at least 20 species, with 68% missing data.

Quartet concordance values

The distributions of concordance values were similar for the crown-ML tree (mean = 0.395, median = 0.35) and the crown-SVD tree (mean = 0.325, median = 0.28) (Fig S2).

Analyses on crown-SVD tree

Results from analyses on the crown-SVD tree are consistent with the analyses on the crown-ML presented in the main text. Table S5 reports model marginal likelihoods and confidence intervals for inferred parameters of BiSSE and HiSSE models. Figure S8 shows parameter estimates and ancestral character reconstructions for the full BiSSE model (B1) and the full HiSSE model (H1). The FiSSE statistic finds the inverse equal splits speciation rate for bee syndrome species is greater than that for hummingbird syndrome species ($\Lambda_0 = 2.48$, $\Lambda_1 = 1.91$), a difference that is significantly larger than expected if diversification rate is not associated with pollination syndrome ($p = 0.015$). Figure S9 shows calculated metrics on simulated trees. Figure S10 shows calculated equilibrium proportions of hummingbird syndrome species based on BiSSE model parameters.

3. Supplemental Tables

Table S1. Character data used in state-dependent diversification analyses on crown clade. Assignments of hummingbird specialist vs. bee syndrome/ despecialized ("partial") species follows (Wilson et al. 2007).

**Species**	**Assigned syndrome**
Penstemon absarokensis	bee
P. acuminatus	bee
P. alamosensis	hummingbird
P. albomarginatus	bee
P. ambiguous	bee
P. ammophilus	bee
P. amphorellae	bee
P. angustifolius	bee
P. arenicola	bee
P. barbatus	hummingbird
P. bicolor	bee
P. bracteatus	bee
P. buckleyi	bee
P. campanulatus	bee
P. cardinalis	hummingbird
P. carnosus	bee
P. caryi	bee
P. cedrosensis	hummingbird
P. centranthifolius	hummingbird
P. clevelandii	bee
P. clutei	bee
P. comarrhenus	bee
P. compactus	bee
P. confusus	bee
P. cyananthus	bee
P. cyaneus	bee
P. cyanocaulis	bee
P. cyathophorus	bee
P. deaveri	bee
P. eatonii	hummingbird
P. eximeus	bee
P. fasciculatus	hummingbird
P. fendleri	bee
P. floridus	bee
P. flowersii	bee

P. fremontii	bee
P. fruticiformis	bee
P. gentryi	bee
P. gibbensii	bee
P. glaber	bee
P. grandiflorus	bee
P. grinnellii	bee
P. hallii	bee
P. harringtonii	bee
P. hartwegii	bee
P. havardii	hummingbird
P. haydenii	bee
P. henricksonii	hummingbird
P. hidalgensis	bee
P. immanifestus	bee
P. incertus	bee
P. isophyllus	bee
P. kunthii	hummingbird
P. labrosus	hummingbird
P. laevis	bee
P. leiophyllus	bee
P. lemhiensis	bee
P. lentus	bee
P. longiflorus	bee
P. mensarum	bee
P. mucronatus	bee
P. murrayanus	hummingbird
P. navajoa	bee
P. neomexicanus	bee
P. nitidus	bee
P. nudiflorus	bee
P. osterhoutii	bee
P. pachyphyllus	bee
P. palmeri	bee
P. parryi	bee
P. parvus	bee
P. patens	bee
P. payettensis	bee
P. paysoniorum	bee

P. penlandii	bee
P. pennellianus	bee
P. perfoliatus	bee
P. petiolatus	bee
P. pseudoputus	bee
P. pseudospectabilis	bee
P. putus	bee
P. roseus	bee
P. salterius	bee
P. saxosorum	bee
P. scariosus	bee
P. secundiflorus	bee
P. skutchii	hummingbird
P. speciosus	bee
P. spectabilis	bee
P. stephensii	bee
P. strictiformis	bee
P. strictus	bee
P. subglaber	bee
P. subulatus	hummingbird
P. superbus	hummingbird
P. tepicensis	bee
P. thurberi	bee
P. tidestromii	bee
P. uintahensis	bee
P. utahensis	hummingbird
P. virgatus	bee
P. wardii	bee
P. wislizenii	hummingbird
P. wrightii	hummingbird

Table S2. SSE models analyzed using MCMC in RevBayes for the crown-ML tree. Marginal log-likelihoods (lnL) of each model are given along with estimates of diversification rates (r) and transition rates (q). The maximum a posteriori (MAP) estimate is provided, along with 95% confidence intervals (CI). Here 0 denotes bee syndrome, 1 denotes hummingbird syndrome, A and B denote hidden character states.

model	marginal lnL	diversification rates MAP (95% CI)	transition rates MAP (95% CI)
B1<br>$r_0 \neq r_1$, $q_{01} \neq q_{10}$	-380.395	r_0 = 2.61 (2.12 – 3.2)<br>r_1 = -0.01 (-1.61 – 0.92)	q_{01} = 0.46 (0.28 – 0.77)<br>q_{10} = 0.05 (0.01 – 1.06)
B2<br>$r_0 \neq r_1$, $q_{01} = q_{10}$	-380.39	r_0 = 2.6 (2.13 – 3.22)<br>r_1 = -0.02 (-1.39 – 1.01)	$q_{01} = q_{10}$ = 0.48 (0.28 – 0.83)
B3<br>$r_0 \neq r_1$, $q_{10} = 0$	-380.2762	r_0 = 2.6 (2.12 – 3.21)<br>r_1 = -0.08 (-1.8 – 0.82)	q_{01} = 0.42 (0.27 – 0.73)<br>q_{10} = 0
B4<br>$r_0 = r_1$, $q_{01} \neq q_{10}$	-384.7861	$r_0 = r_1$ = 2.36 (1.92 – 2.89)	q_{01} = 0.39 (0.24 – 0.65)<br>q_{10} = 0.09 (0.01 – 1.54)
H1<br>$r_{0A} \neq r_{1A} \neq r_{0B} \neq r_{1B}$<br>$q_{01} \neq q_{10} \neq q_{AB} \neq q_{BA}$	-378.1629	r_{0A} = 3.35 (2.45 – 5.09)<br>r_{1A} = 0.04 (-4.02 – 1.35)<br>r_{0B} = 0.22 (-1 – 1.53)<br>r_{1B} = -0.08 (-3.52 – 1.55)	q_{01} = 0.46 (0.29 – 0.8)<br>q_{10} = 0.05 (0.004 – 1.04)<br>q_{AB} = 1.58 (0.11 – 3.41)<br>q_{BA} = 0.03 (0.004 – 0.79)
CID2<br>$r_{0A} = r_{1A} \neq r_{0B} = r_{1B}$<br>$q_{01} \neq q_{10} \neq q_{AB} \neq q_{BA}$	-380.9433	$r_{0A} = r_{1A}$ = 3.48 (2.41 – 2.92)<br>$r_{0B} = r_{1B}$ = 0.19 (-0.63 – 1.11)	q_{01} = 0.38 (0.23 – 0.66)<br>q_{10} = 0.1 (0.03 – 1.58)<br>q_{AB} = 1.97 (0.48 – 3.65)<br>q_{BA} = 0.03 (0.005 – 0.64)

Table S3. SSE models analyzed using MCMC in RevBayes for the crown-ML-cropped tree. Marginal log-likelihoods (lnL) of each model are given along with estimates of diversification rates (r) and transition rates (q). The maximum a posteriori (MAP) estimate is provided, along with 95% confidence intervals (CI). Here 0 denotes bee syndrome, 1 denotes hummingbird syndrome, A and B denote hidden character states.

model	marginal lnL	diversification rates MAP (95% CI)	transition rates MAP (95% CI)
B1<br>$r_0 \neq r_1$, $q_{01} \neq q_{10}$	-383.9136	r_0 = 3.41 (2.7 – 4.31)<br>r_1 = -0.07 (-2.37 – 1.16)	q_{01} = 0.61 (0.37 – 1.03)<br>q_{10} = 0.09 (0.009 – 1.48)
B2<br>$r_0 \neq r_1$, $q_{01} = q_{10}$	-384.2393	r_0 = 3.33 (2.69 – 4.32)<br>r_1 = 0.001 (-2.03 – 1.26)	$q_{01} = q_{10}$ = 0.61 (0.37 – 1.11)
B3<br>$r_0 \neq r_1$, $q_{10} = 0$	-383.5977	r_0 = 3.35 (2.71 – 4.3)<br>r_1 = -0.06 (-2.92 – 0.9)	q_{01} = 0.57 (0.35 – 1.0)<br>q_{10} = 0
B4<br>$r_0 = r_1$, $q_{01} \neq q_{10}$	-389.67	$r_0 = r_1$ = 3.01 (2.42 – 3.69)	q_{01} = 0.51 (0.3 – 0.9)<br>q_{10} = 0.31 (0.04 – 2.58)
H1<br>$r_{0A} \neq r_{1A} \neq r_{0B} \neq r_{1B}$<br>$q_{01} \neq q_{10} \neq q_{AB} \neq q_{BA}$	-375.4249	r_{0A} = 3.58 (2.82 – 4.84)<br>r_{1A} = 0.01 (-2.87 – 1.34)<br>r_{0B} = 0.11 (-2.99 – 3.05)<br>r_{1B} = 0.04 (-5.08 – 3.73)	q_{01} = 0.59 (0.37 – 1.04)<br>q_{10} = 0.07 (0.01 – 1.46)<br>q_{AB} = 0.13 (0.02– 2.08)<br>q_{BA} = 0.08 (0.004 – 1.46)
CID2<br>$r_{0A} = r_{1A} \neq r_{0B} = r_{1B}$<br>$q_{01} \neq q_{10} \neq q_{AB} \neq q_{BA}$	-380.9433	$r_{0A} = r_{1A}$ = 3.48 (2.41 – 4.92)<br>$r_{0B} = r_{1B}$ = 0.19 (-0.63 – 1.11)	q_{01} = 0.38 (0.23 – 0.66)<br>q_{10} = 0.1 (0.03 – 1.58)<br>q_{AB} = 1.97 (0.48 – 3.65)<br>q_{BA} = 0.03 (0.005 – 0.64)

Table S4. Marginal log-likelihoods (lnL) of selected SSE models estimated for the crown-ML tree with 5% and 7.5% of total length truncation.

Tree	Model	Marginal lnL
5% truncation	B1	-365.7172
5% truncation	H1	-364.4734
5% truncation	CID2	-368.0895
7.5% truncation	B1	-359.5473
7.5% truncation	H1	-359.0289
7.5% truncation	CID2	-362.6299

Table S5. SSE models analyzed using MCMC in RevBayes for the crown-SVD tree. Marginal log-likelihoods (lnL) of each model are given along with estimates of diversification rates (r) and transition rates (q). The maximum a posteriori (MAP) estimate is provided, along with 95% confidence intervals (CI). Here 0 denotes bee syndrome, 1 denotes hummingbird syndrome, A and B denote hidden character states.

model	marginal lnL	diversification rates MAP (95% CI)	transition rates MAP (95% CI)
B1<br>$r_0 \neq r_1$, $q_{01} \neq q_{10}$	-383.9136	r_0 = 2.53 (2.02 – 3.06)<br>r_1 = -0.03 (-1.23 – 0.77)	q_{01} = 0.43 (0.27 – 0.74)<br>q_{10} = 0.04 (0.004 – 0.9)
B2<br>$r_0 \neq r_1$, $q_{01} = q_{10}$	-384.2393	r_0 = 2.46 (2.03 – 3.07)<br>r_1 = -0.01 (-1.12 – 0.79)	q_{01} = q_{10} = 0.45 (0.27 – 0.80)
B3<br>$r_0 \neq r_1$, $q_{10} = 0$	-383.5977	r_0 = 2.56 (2.03 – 3.06)<br>r_1 = -0.02 (-1.3 – 0.65)	q_{01} = 0.42 (0.26 – 0.69)<br>q_{10} = 0
B4<br>$r_0 = r_1$, $q_{01} \neq q_{10}$	-389.67	r_0 = r_1 = 2.25 (1.81 – 2.72)	q_{01} = 0.38 (0.22 – 0.63)<br>q_{10} = 0.13 (0.03 – 1.4)
H1<br>$r_{0A} \neq r_{1A} \neq r_{0B} \neq r_{1B}$<br>$q_{01} \neq q_{10} \neq q_{AB} \neq q_{BA}$	-375.4249	r_{0A} = 4.44 (3.22 – 6.3)<br>r_{1A} = -0.09 (-6.29 – 1.8)<br>r_{0B} = 0.07 (-0.57 – 0.76)<br>r_{1B} = -0.04 (-1.9 – 0.97)	q_{01} = 0.42 (0.27 – 0.76)<br>q_{10} = 0.04 (0.005 – 0.96)<br>q_{AB} = 3.1 (1.66 – 4.81)<br>q_{BA} = 0.02 (0.003 – 0.5)
CID2<br>$r_{0A} = r_{1A} \neq r_{0B} = r_{1B}$<br>$q_{01} \neq q_{10} \neq q_{AB} \neq q_{BA}$	-380.9433	r_{0A} = r_{1A} = 4.34 (3.03 – 6.11)<br>r_{0B} = r_{1B} = 0.09 (-0.47 – 0.64)	q_{01} = 0.37 (0.21 – 0.62)<br>q_{10} = 0.08 (0.01 – 1.40)<br>q_{AB} = 3.25 (1.99– 5.01)<br>q_{BA} = 0.02 (0.002 – 0.44)

4. Supplemental figures

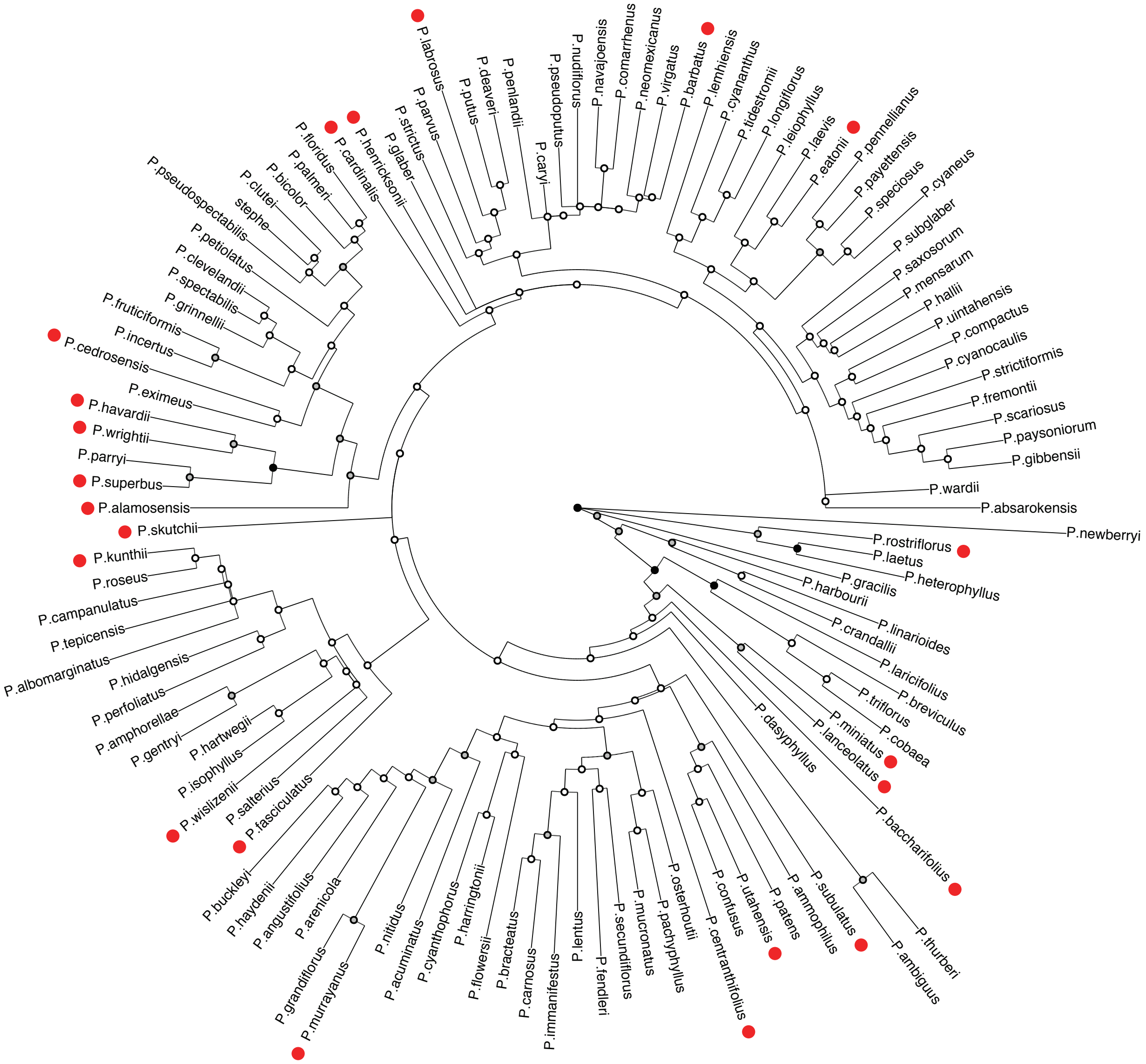

Fig. S1. Species tree inferred for the full sample of 120 *Penstemon* species using SVDquartets. Hummingbird specialist species are marked with red dots. Nodes are colored according to bootstrap support values: black is greater than 95%, grey is 75–95%, white is less than 75%. Branch lengths were estimated using a constrained tree search in IQ-TREE under a GTR + Γ model.

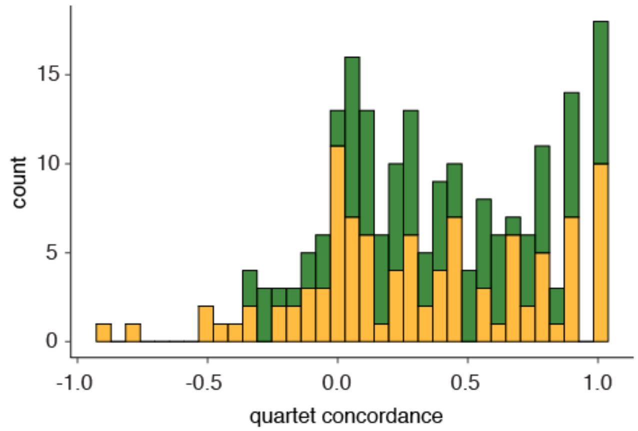

Fig. S2. Distributions of quartet concordance metric values calculated for each node. Dark green: crown-ML tree, orange: crown-SVD tree.

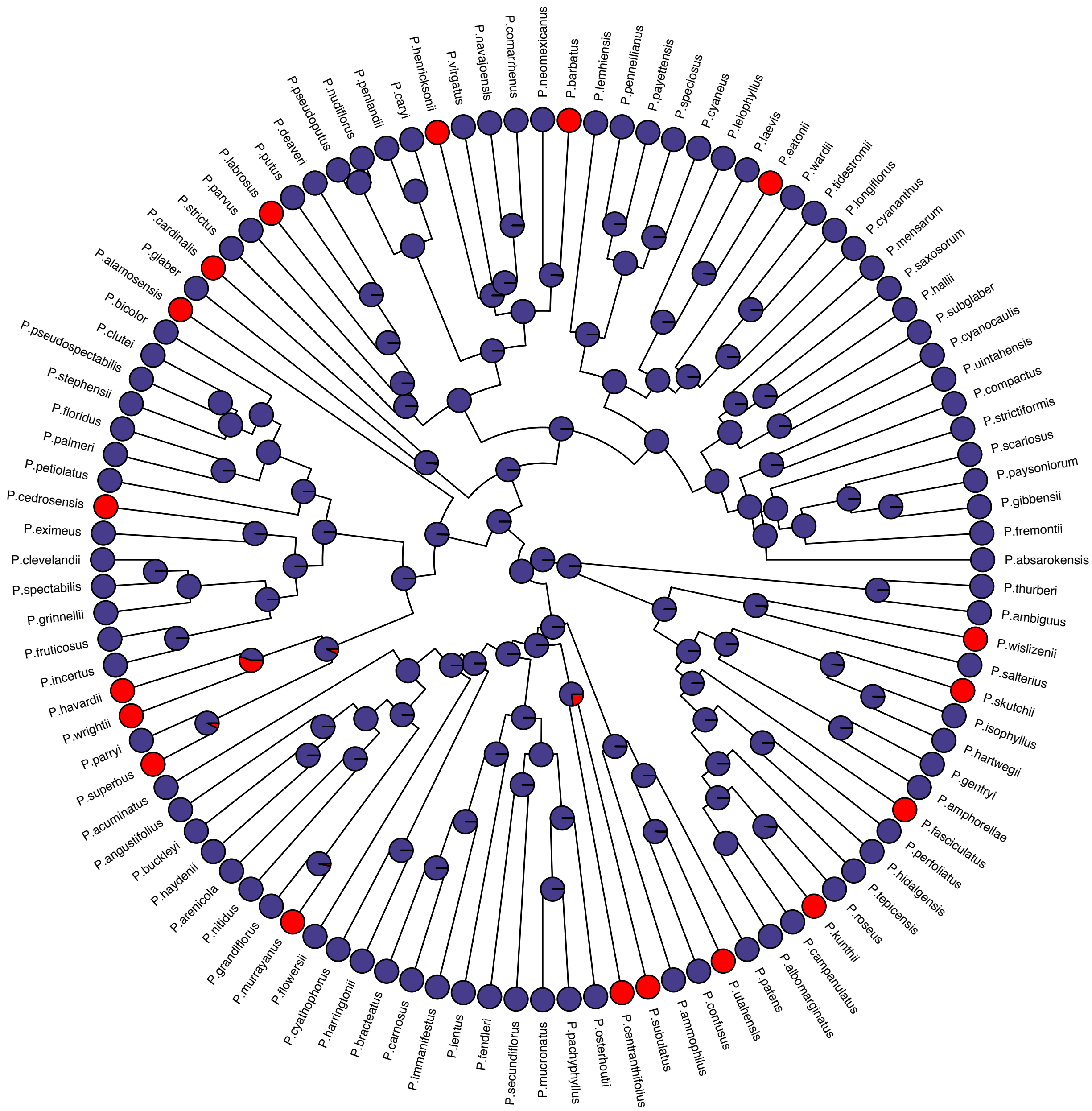

Fig. S3. Ancestral character states for the crown-ML tree under the B1 BiSSE model, showing taxon names. Blue = bee syndrome, red = hummingbird syndrome.

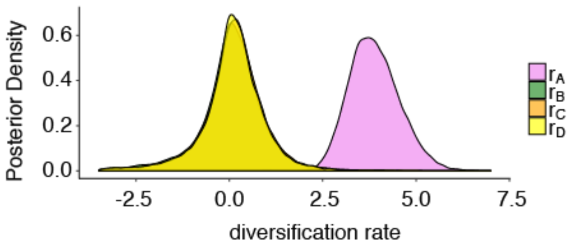

Fig. S4. Posterior densities of net diversification rates for the CID4 model estimated from crown-ML tree. Rates for states B, C, and D are nearly completely overlapping.

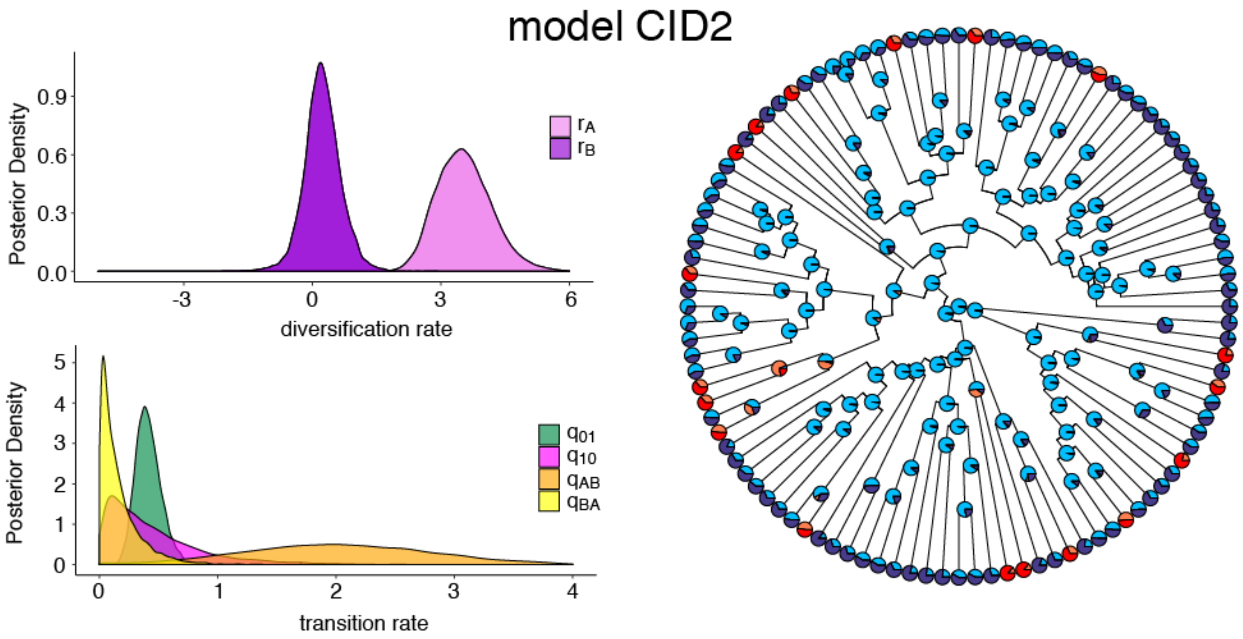

Fig. S5. Posterior distributions of parameter values and ancestral character states under the CID2 model estimated from crown-ML tree. In ancestral reconstruction, light blue = 0A, light red = 1A, dark blue = 0B, dark red = 1B.

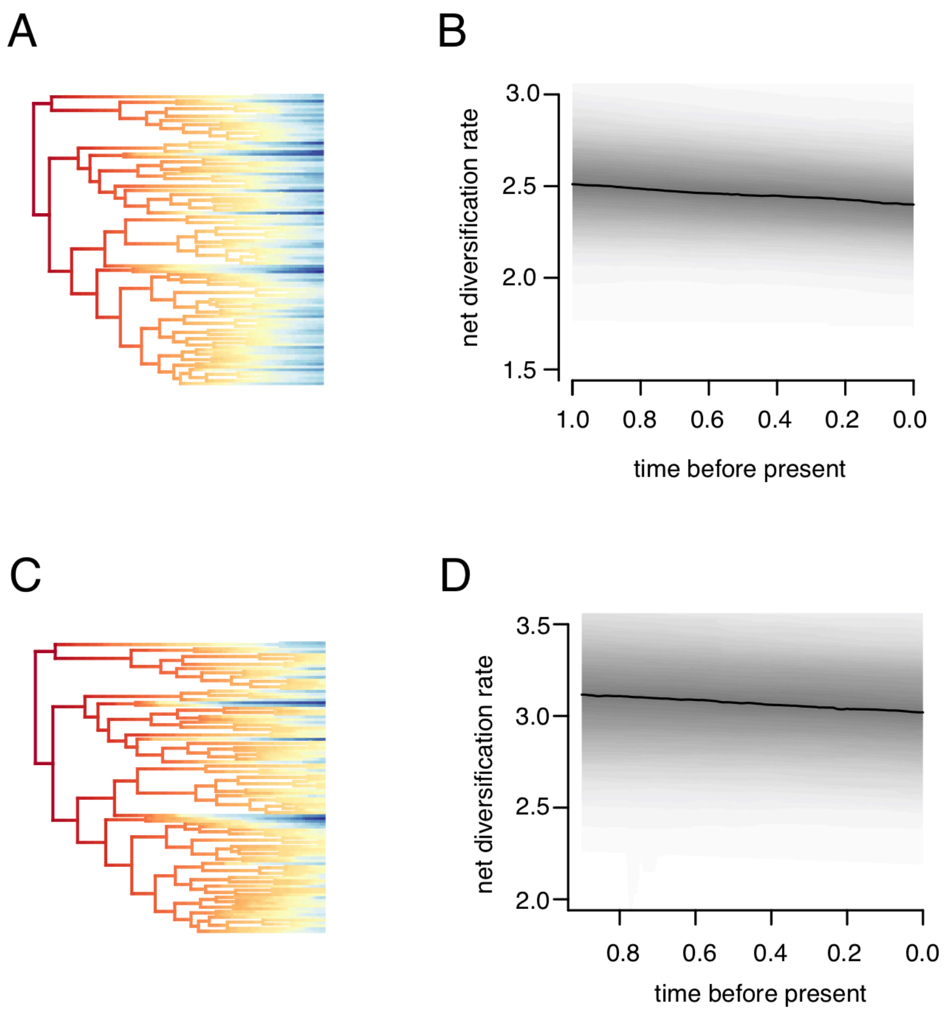

Fig. S6. Character-independent patterns of diversification in the *Penstemon* crown clade. Panels A and C: visualization of model-averaged net diversification across the tree inferred using BAMM (red = higher diversification rate, blue = lower rate). Panels B and D: model-averaged net diversification rate inferred using BAMM as a function of time. Plots are shown for the crown-ML tree (panels A and B) and the crown-ML-cropped tree (panels C and D). The crown-ML and crown-ML-cropped trees each display a single diversification regime with no lineage-specific shifts in diversification rate, according to Bayes factor model comparison implemented in BAMMtools. In each of these trees, the net diversification rate slows down through time within the single regime. In panel B the slowdown is from 2.5105–2.400; in panel D the slowdown is from 3.1163–3.0193.

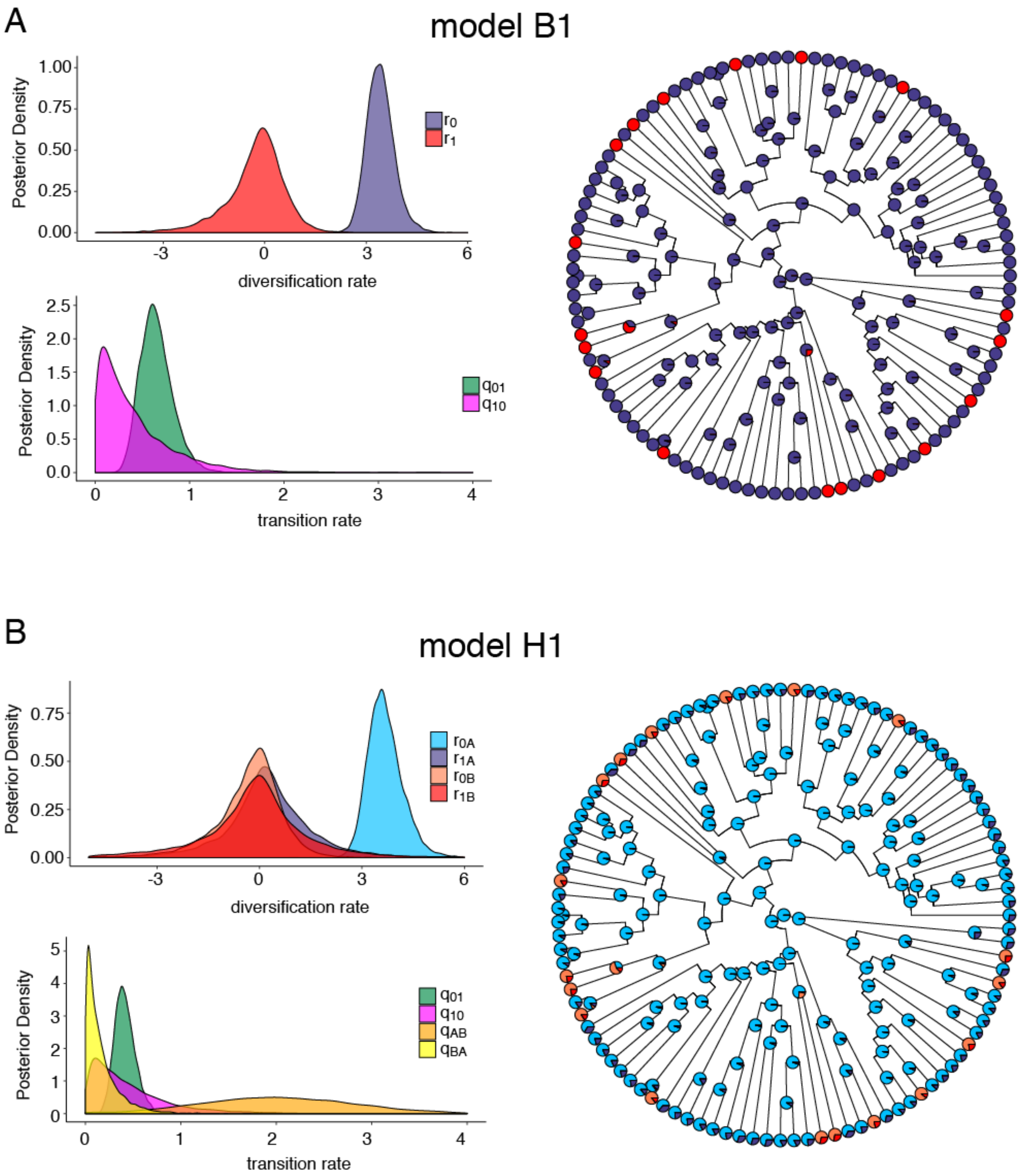

Fig. S7. Posterior parameter distributions and ancestral character states for models estimated from the crown-ML-cropped tree. A: Estimates for full BiSSE model B1, B: estimates for full HiSSE model H1. For ancestral states, colors correspond to the posterior probabilities for a given state. In panel A, blue = bee syndrome, red = hummingbird syndrome. In panel B, light blue = state 0A, light red = state 1A, blue = state 0B, red = state 1B.

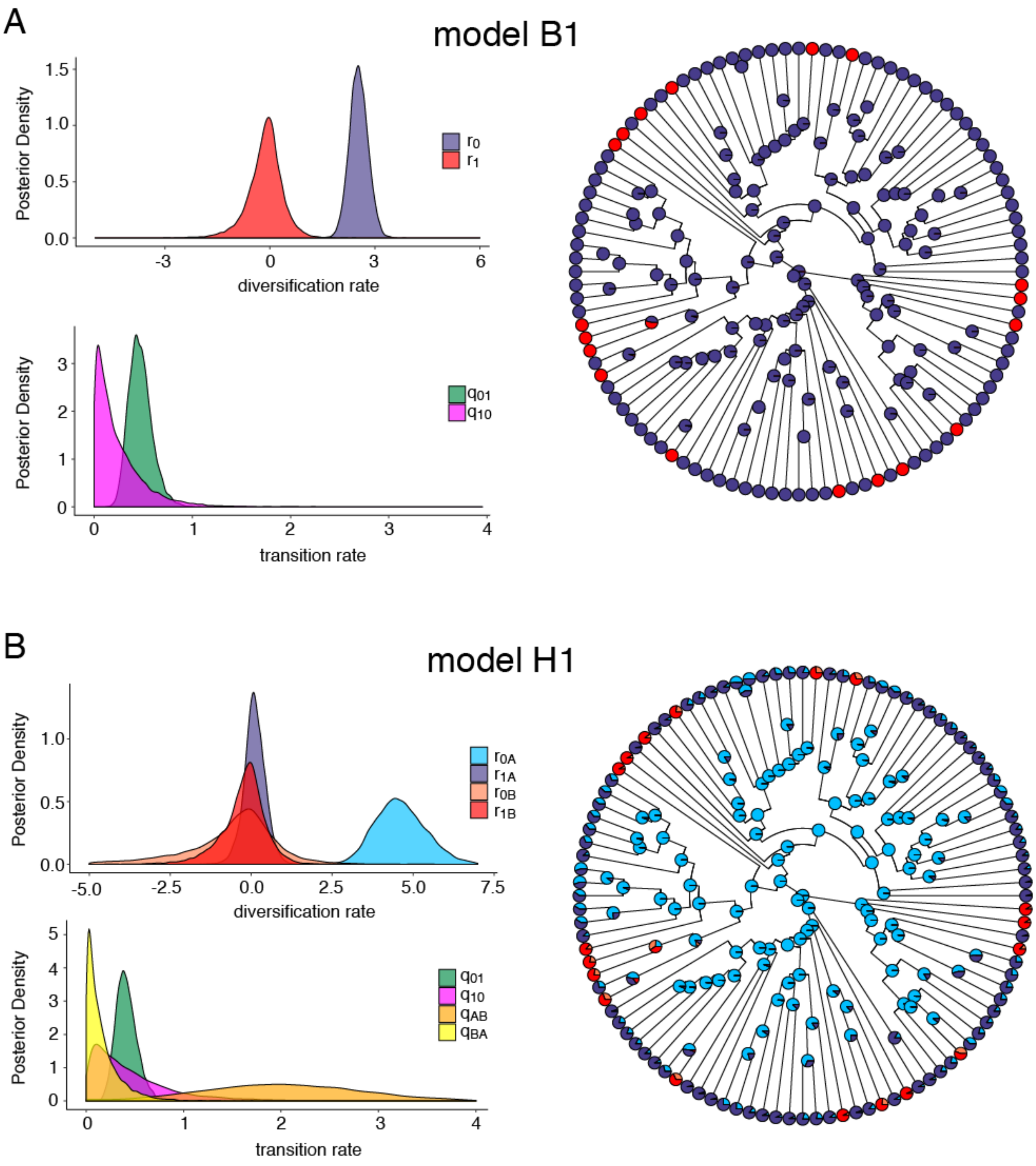

Fig. S8. Posterior parameter distributions and ancestral character states for models estimated from the crown-SVD tree. A: Estimates for full BiSSE model B1, B: estimates for full HiSSE model H1. For ancestral states, colors correspond to the posterior probabilities for a given state. In panel A, blue = bee syndrome, red = hummingbird syndrome. In panel B, light blue = state 0A, light red = state 1A, blue = state 0B, red = state 1B.

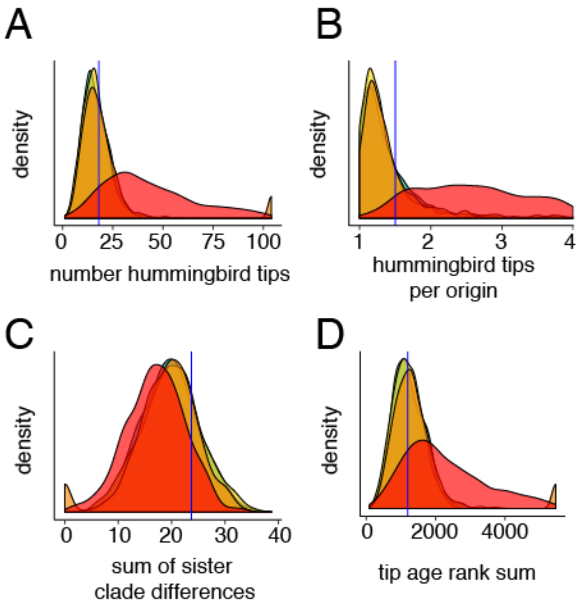

Fig. S9. Distributions of metrics for 1000 simulated trees of 104 species for selected models estimated from the crown-SVD tree. Green: model B1 (full BiSSE), yellow: model B2 (equal transition rates), orange: model B3 (unidirectional transitions), and red: model B4 (equal diversification rates). Blue vertical lines indicate observed value for the empirical dataset.

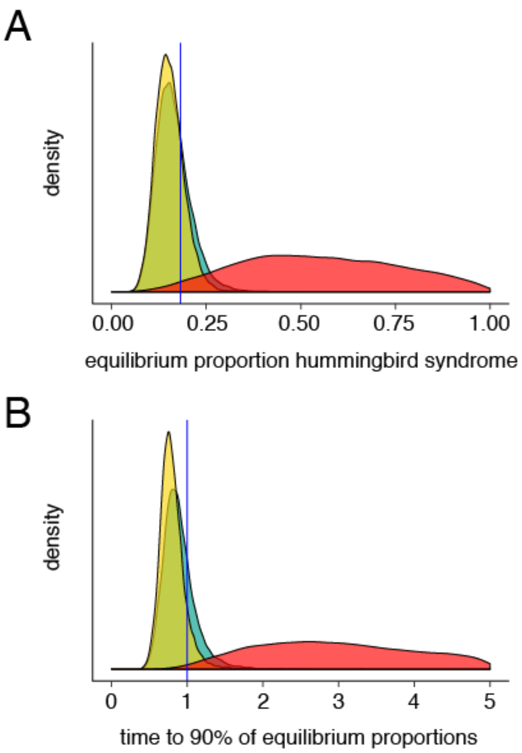

Fig. S10. Results from equilibrium calculations for the crown-SVD tree. A: Distributions of the proportion of hummingbird syndrome species at equilibrium, blue vertical line indicates current proportion of hummingbird syndrome species in the crown clade. B: Distributions of the time taken for the proportion hummingbird syndrome species to reach 90% of the expected equilibrium proportion. Green: model B1 (full BiSSE), yellow: model B2 (equal transition rates), and red: model B4 (equal diversification rates)

Supplemental References

Beaulieu, J. M. and B. C. O'Meara. 2016. Detecting hidden diversification shifts in models of trait-dependent speciation and extinction. Systematic biology 65:583-601.

Bouckaert, R., J. Heled, D. Kühnert, T. Vaughan, C.-H. Wu, D. Xie, M. A. Suchard, A. Rambaut, and A. J. Drummond. 2014. BEAST 2: a software platform for Bayesian evolutionary analysis. PLoS computational biology 10:e1003537.

Bromham, L., X. Hua, and M. Cardillo. 2015. Detecting macroevolutionary self-destruction from phylogenies. Systematic biology 65:109-127.

Chifman, J. and L. Kubatko. 2014. Quartet inference from SNP data under the coalescent model. Bioinformatics 30:3317-3324.

Eaton, D. A., E. L. Spriggs, B. Park, and M. J. Donoghue. 2017. Misconceptions on missing data in RAD-seq phylogenetics with a deep-scale example from flowering plants. Systematic Biology 66:399-412.

Hoang, D. T., O. Chernomor, A. von Haeseler, B. Q. Minh, and L. S. Vinh. 2017. UFBoot2: improving the ultrafast bootstrap approximation. Molecular biology and evolution 35:518-522.

Höhna, S., M. J. Landis, and T. A. Heath. 2017. Phylogenetic inference using revbayes. Current protocols in bioinformatics 57:6.16. 11-16.16. 34.

Höhna, S., M. J. Landis, T. A. Heath, B. Boussau, N. Lartillot, B. R. Moore, J. P. Huelsenbeck, and F. Ronquist. 2016. RevBayes: Bayesian phylogenetic inference using graphical models and an interactive model-specification language. Systematic Biology 65:726-736.

Huang, H. and L. L. Knowles. 2014. Unforeseen consequences of excluding missing data from next-generation sequences: simulation study of RAD sequences. Systematic biology 65:357-365.

Kass, R. E. and A. E. Raftery. 1995. Bayes factors. Journal of the american statistical association 90:773-795.

Maddison, W. P., P. E. Midford, and S. P. Otto. 2007. Estimating a binary character's effect on speciation and extinction. Systematic biology 56:701-710.

Miller, M. A., W. Pfeiffer, and T. Schwartz. 2010. Creating the CIPRES Science Gateway for inference of large phylogenetic trees. Pp. 1-8. 2010 gateway computing environments workshop (GCE). Ieee.

Nguyen, L.-T., H. A. Schmidt, A. von Haeseler, and B. Q. Minh. 2014. IQ-TREE: a fast and effective stochastic algorithm for estimating maximum-likelihood phylogenies. Molecular biology and evolution 32:268-274.

Rabosky, D. L. 2014. Automatic detection of key innovations, rate shifts, and diversity-dependence on phylogenetic trees. PloS one 9:e89543.

Rabosky, D. L. and E. E. Goldberg. 2015. Model inadequacy and mistaken inferences of trait-dependent speciation. Systematic Biology 64:340-355.

Rabosky, D. L. and E. E. Goldberg. 2017. FiSSE: A simple nonparametric test for the effects of a binary character on lineage diversification rates. Evolution 71:1432-1442.

Rabosky, D. L., M. Grundler, C. Anderson, P. Title, J. J. Shi, J. W. Brown, H. Huang, and J. G. Larson. 2014. BAMM tools: an R package for the analysis of evolutionary dynamics on phylogenetic trees. Methods in Ecology and Evolution 5:701-707.

Rambaut, A., A. J. Drummond, D. Xie, G. Baele, and M. A. Suchard. 2018. Posterior summarisation in Bayesian phylogenetics using Tracer 1.7. Syst. Biol 10.

Swofford, D. 2003. PAUP* phylogenetic analysis using parsimony (*and other methods), version 4. Sinauer, Sunderland, Massachusetts, USA.

Wessinger, C. A., C. C. Freeman, M. E. Mort, M. D. Rausher, and L. C. Hileman. 2016. Multiplexed shotgun genotyping resolves species relationships within the North American genus Penstemon. American Journal of Botany 103:912-922.

Wilson, P., A. D. Wolfe, W. S. Armbruster, and J. D. Thomson. 2007. Constrained lability in floral evolution: counting convergent origins of hummingbird pollination in Penstemon and Keckiella. New Phytologist 176:883-890.
Wolfe, A. D., C. P. Randle, S. L. Datwyler, J. J. Morawetz, N. Arguedas, and J. Diaz. 2006. Phylogeny, taxonomic affinities, and biogeography of Penstemon (Plantaginaceae) based on ITS and cpDNA sequence data. American Journal of Botany 93:1699-1713.
Zhou, X., X.-X. Shen, C. T. Hittinger, and A. Rokas. 2017. Evaluating fast maximum likelihood-based phylogenetic programs using empirical phylogenomic data sets. Molecular biology and evolution 35:486-503.
